# The impact of caring for children on women’s research output: A retrospective cohort study

**DOI:** 10.1371/journal.pone.0214047

**Published:** 2019-03-21

**Authors:** Lauren Sewell, Adrian G. Barnett

**Affiliations:** School of Public Health and Social Work, Queensland University of Technology, Brisbane, Australia; Universidad de las Palmas de Gran Canaria, SPAIN

## Abstract

We used a retrospective cohort study to measure the impact of caring for children on female Australian researchers. Our aim was to see whether caring for children was associated with reduced outputs and collaboration. Women were randomly selected for inclusion if they published a first author paper in one of three Australian journals during 2007 to 2015, women who did not publish during this time were not included. One-hundred and sixty women were approached and 95 (59%) completed a survey on their history of caring for children. Two key outcomes were the women’s publication and citation counts, which were accessed from *Scopus*. We also examined the number of authors, affiliations and countries on their published papers, as a reduction in these numbers could indicate an impaired ability to collaborate. We examined the probability of being first or last author as a measure of esteem. There was a small increase in publication counts after the first child that was reversed after the second child. Average citations counts declined after children, particularly after the second child. There was some evidence of a reduced collaboration with overseas collaborators after the first child. The probability of being the last author increased after the second child. Three women were identified as statistically influential and all three had children and were in the top 10% of overall publications and citations. After removing these women the estimated changes in outcomes were noticeably different for most of the outcomes. The repeated presence of statistically influential women shows that it may be impossible to find an “average impact” of caring for children when considering research output. Adjustments may need to be made individually, with women explaining how caring for children has altered their career.

## Introduction

In Australia, the two primary agencies responsible for awarding research funding are the National Health and Medical Research Council (NHMRC) and the Australian Research Council (ARC) [1]. Although the ARC and NHMRC evaluate grant applications differently, both have been criticised for the heavy weighting given to track record [1]. An interviewee in a study conducted by Mow [1] on grant funding and peer review said,

“excellent research is not getting funded. The old boys’ club is growing as track record becomes more important for the selectors because of the weight given to the selection criteria.”

The ARC and NHMRC always allow applicants to detail career disruptions which may have impacted their research performance, including child birth and carers’ responsibilities [2, 3]. Both agencies acknowledge the potential impact of career disruptions, and assess outputs relative to opportunity, but it is unclear how exactly this is achieved. It is also not clear whether any adjustments are consistently applied as the assessment of disruption is made by peer reviewers who often have different views and experiences of the impacts of caring for children [4].

There is limited research which quantifies the impact of caring for children on research output, and filling this gap could inform the process of adjusting track record. Previous research has focused on the differences between men and women and the factors that initiate and perpetuate any inequalities, including: family commitments, organisational context and culture, and academic rank.

Key studies in the field have conflicting results. While some studies have shown that children have no impact on output [5, 6], other studies have shown that children have either a negative [7] or positive impact on output [8]. A review of the literature comparing male and female researchers also found contradictory evidence with no clear-cut picture of the main sources of the gender gap in research output [9].

Methodological weaknesses have been suggested as the reason for the inconsistent findings of previous studies [10]. Previous research has been criticised for measuring research output over short time spans [8, 11]; capturing parental status as a simple binary variable (Yes/No) without considering when child-care began or the number and ages of the children [12]; and recruiting participants from a single academic field, many of which have a disproportionate representation of males [13].

A 2010 study by Hunter and Leahey sought to overcome many of the shortcomings of past research. Hunter and Leahey recruited participants from the fields of linguistics and sociology due to lower levels of gender segregation [10]. The number and ages of each child in a woman’s care was recorded, and research output was calculated over a woman’s career lifetime using publication counts, and visibility measured using citation counts and journal impact-weighted publications. Their results showed that, while children had an initial positive impact on output, they had a negative long-term impact on output growth. Their results also showed that both men and women experienced a reduction in citation numbers after the birth of a child, however this impact was only sustained over the long-term for women. They suggested a number of possible causes of women’s reduced long-term visibility, including: reduced publishing, difficulty maintaining collegial networks, and time limitations precipitating sacrifices in quality.

A number of Australian studies have examined the relationship between children and research output. An Australian paper published in 2012 by Klocker and Drozdzewski reported on women’s opinion of the phrase “relative to opportunity” and the extent to which they felt it made a difference to their career, including their likelihood of promotion. Klocker and Drozdzewski asked female researchers how many papers they felt a child was worth. The question sparked controversy, with one researcher stating,

“You cannot quantify output and productivity like that … we need to take a more holistic look at the CVs to suss out the candidates and what they have to offer in a range of areas” [14].

Women’s prediction of how many papers a child was worth varied from 1–2 to 3–4 papers per year on average. Overall, women felt that research output was not assessed fairly in the context of family commitments. They wrote “relative to opportunity” was “largely perceived as a tokenistic gesture put on forms and never taken into account by the people who make decisions and evaluate work” [14].

Our study aims to address many of the limitations of previous research. Similarly to Hunter and Leahey [10], we evaluate women’s output over their entire career and measure both the output and research visibility of women using publications and citations. We build on Hunter and Leahey’s “children” variable by examining a time-varying impact of caring for children. We use publication counts to assess research output as this measure remains one of the most widely used and accepted measures [13]. We use citation counts and these are a common proxy of research quality and impact [10, 15]. A review of what factors influence citation counts found three papers which showed quality was a “strong predictor” of citation counts and one paper found quality to be a “weak predictor” [16]. We note that citation counts are by no means a perfect measure of article quality, and that there are many other predictors of citation counts including the authors’ nationality, number of authors, number of references, and recency of the references [16].

We used three measures of research collaboration in an attempt to investigate a potential impact on women’s networks after caring for children. These were the number of authors per paper, the number of affiliations per paper, and the number of co-author countries outside Australia. If women caring for children had difficulty maintaining networks, then we would expect these numbers to decline after caring commenced.

## Materials and methods

### Study design

We used a retrospective cohort study using a randomly selected sample of Australian female researchers in health and medicine. The outputs of women with children was compared with the outputs of women without children over each woman’s career history. Career history was defined as the time from their first publication to the end of 2015, and research output was assessed as a woman’s annual publication and citation counts.

The controls were women who had not cared for children. Due to the longitudinal study design, women with children also acted as their own control as their research output was compared before and after caring for a child. This helped control for characteristics that might impact on output, e.g., non-native English speaker.

A cohort design was used as we felt that women’s research output could only be accurately assessed by observing their entire career trajectory rather than their performance at a moment in time. Previous studies have been criticised for assessing output over short time spans, and there has been a call for longitudinal studies [11].

### Setting

Women recruited were researchers who had published in one of three Australian medical journals (see next section for details). Recruitment was between October 2015 and December 2015. Email addresses of sampled women were sourced from the original publication, or through other journal publications, and/or searches of *Google* and *LinkedIn*.

In January 2016, participants were sent an email requesting them to complete a short online survey. Women who did not respond to the email were sent a reminder email after two weeks. Women who had not completed the survey after a further two weeks were mailed a paper survey.

The survey asked participants: 1) if they had ever cared for a child, including biological/foster, adoptive, and or step-child, 2) the number of children they had cared for, 3) for each child, the date care commenced and the type of care provided, e.g., biological parent or step-parent (see S1 Appendix for survey). The survey also included a free-text section where participants had the opportunity to comment on their experience of working while caring for children.

The definition of “child” was kept broad and included biological/adoptive parent, step parent, foster parent, and/or legal guardian/other. These classifications were based on the Australian Bureau of Statistics who include categories for natural, step, and foster children. Adopted children are categorised together with natural children by the Australian Bureau of Statistics [17].

### Participants

Participants were a random sample of 203 women who published a first author paper in the Medical Journal of Australia, Australian and New Zealand Journal of Public Health, or the Australian and New Zealand Journal of Obstetrics and Gynaecology between 2007 and 2015. Women who did not publish a first author paper during these years were not included. Two issues per journal per year were randomly sampled to give a list of authors. These journals were chosen as three prominent Australia-based journals that would include a high proportion of papers from Australian authors. The starting year of 2007 was chosen to give a reasonable amount of follow-up time whilst also reducing the chance of not being able to contact women due to address changes. Only women residing in Australia at the time of publication were included. No other inclusion criteria were applied.

Four-hundred and sixty-five women were assessed for eligibility and 262 were excluded due to not meeting the inclusion criteria (see S1 Fig for flow diagram of participant recruitment). A further 43 women were not recruited because contact details could not be found. The remaining 160 eligible participants were sent the email in January 2016 and 95 responded.

Multiple efforts were made to source women’s contact details, but as the start of the recruitment period was nine years prior to data collection, contact details for some women were difficult to obtain. Women who are no longer active in research are a particularly important group to capture, as they may have left due to child-caring responsibilities. Analysing these women is also important in reducing potential biases of only analysing the output of women who are research-active. To examine this potential bias, we compared the research output of women who did and did not respond to the survey using t-tests for total citation counts, publication counts and year of first publication.

The study was approved by the Queensland University of Technology Human Ethics Committee. Women provided their consent to participate in the online survey (S1 Appendix).

### Research output data

Research output was measured using annual publication and citation counts. Counts per year were recorded from the year of first publication through to December 2015. Publication and citation information over time for each participant was downloaded from *Scopus* and saved to an Excel file. *Scopus* publications include journal papers, conference papers, books and book chapters.

We used publication counts because it is a commonly used measure of research output [10, 13]. It is also highly relevant as both the ARC and NHMRC assess a researcher’s performance based on evidence of research outputs including refereed journal articles [3, 15].

We used citation counts because it has been viewed as an essential measure of research quality and visibility [10] and relevance [18].

A commonly used measure of research output is the H-index, but there is debate about the merits of using the H-index to assess research output [19, 20], therefore we chose not to use it.

### Research collaboration data

For each paper we examined the number of authors and number of affiliations as measures of research collaboration, with higher numbers indicating greater collaboration. We also examined the number of countries listed for each paper’s authors and counted the number of countries outside Australia as a measure of international collaboration. For 461 papers (12%) there was no country data available. We examined every paper between each woman’s first paper and the end of 2015. To focus on standard research papers, we only used papers or reviews, and excluded editorials, errata or letters.

We examined author order, specifically where the researcher was the first or last author as both positions are recognised measures of esteem. For 8 papers (0.2%) there was no author order available as authorship was solely assigned to a group (e.g., “The XYZ Study Team”).

We used the R packages “rscopus” and “bibliometrix” to extract data from *Scopus* directly into R [21, 22].

### Biases and errors

Recall or information bias and selection bias are two common methodological challenges in cohort studies. In order to reduce selection bias, women were randomly selected to participate in the study. Data on publications and citations were sourced from an independent third party therefore eliminating recall bias. We assume there is negligible error in recalling the dates of caring for children.

### Confounders and effect modifiers

This is a preliminary study into the impact of caring for a child on a woman’s research output. Therefore, an in-depth analysis of the impact of all relevant explanatory variables on women’s research output was not performed. Variables that may warrant further investigation, and have the potential to confound or modify the results, or be on the causal pathway between children and research impact, include: a woman’s age, employment status, any change in employment status after caring for a child, and access to childcare. However, this study aims to quantify the impact of caring for a child, rather than detail the causes of this impact.

### Statistical methods

The data were arranged in longitudinal format as the number of citations and publications per year per woman. Each woman’s data started from the year of her first publication, which we defined as the start of her research career. The data ended in 2015, the last year that full citation and publication statistics were available. The key predictor of child status was a binary variable that was zero if the women had no children in that year, and one if she cared for any children in that year (and remained one for the rest of her career). To examine a potential change in the impact of caring for children over time, we added a time since child variable using the year and month of the child’s birth. To examine the impact of a second child we added another binary child variable and another time since child variable. We did not examine three or more children as this only applied to 11% of the sample. See Table A1 in S2 Appendix for an example of the data structure.

Generalised Linear Mixed Models (GLMMs) with random intercepts per woman were used to examine the impact of children on the women’s careers [23]. In the first model (model 1a), citation or publication counts per year were modelled using the time-dependent predictor first child (yes/no). Years since care began for the first child was then added (model 1b). The impact of caring for two children was modelled by adding any second child (yes/no) and a years since variable (models 2a and b; see S2 Appendix for additional information).

As equations the GLMMs for publication counts for the four models are:

$$\begin{array}{ccc} P_{i,t} & ∼ & \text{Poisson}\left(\mu_{i,t}\right),\;i=1,\ldots ,n,\;t=1,\ldots ,l_{i},\\ \text{Model}\;1a:\;\log(\mu_{i,t}) & = & \alpha_{0}+\alpha_{1}y_{i,t}+\alpha_{2}s_{i,t}+\alpha_{3}I\left(b1_{i,t}>0\right)+\gamma_{i}\\ \text{Model}\;1b:\;\log(\mu_{i,t}) & = & \alpha_{0}+\alpha_{1}y_{i,t}+\alpha_{2}s_{i,t}+\alpha_{3}I\left(b1_{i,t}>0\right)+f\left(\alpha_{4},b1_{i,t}\right)+\gamma_{i}\\ \text{Model}\;2a:\;\log(\mu_{i,t}) & = & \alpha_{0}+\alpha_{1}y_{i,t}+\alpha_{2}s_{i,t}+\alpha_{3}I\left(b1_{i,t}>0\right)+\alpha_{4}I\left(b2_{i,t}>0\right)+\gamma_{i},\\ \text{Model}\;2b:\;\log(\mu_{i,t}) & = & \alpha_{0}+\alpha_{1}y_{i,t}+\alpha_{2}s_{i,t}+\alpha_{3}I\left(b1_{i,t}>0\right)+f\left(\alpha_{4},b1_{i,t}\right)\\ & & +\alpha_{5}I\left(b2_{i,t}>0\right)+f\left(\alpha_{6},b2_{i,t}\right)+\gamma_{i},\\ \gamma_{i} & ∼ & \text{Normal}(0,\sigma_{\gamma }^{2}), \end{array}$$

where *i* is the index for the *n* women, and *t* is the index for time which varies in length depending on the career length of each woman (*l*_*i*_). *P*_*i*,*t*_ is the yearly number of publications. *y*_*i*,*t*_ is year (between 1973 and 2015) and this controls for the generally higher number of research outputs over time. *s*_*i*,*t*_ is the year since each woman’s first publication which controls for the expected increase in the number of research outputs with increasing experience. *b*1_*i*,*t*_ is the time in months since caring for the first child began and is zero for women who do not have a child at time *t*. *I*() is an indicator function so that *α*_3_ models the step change in caring for children. The time-dependent change is modelled by the fractional polynomial *f*() (see below). *γ*_*i*_ is a random intercept which controls for the repeated data over time from the same woman. For citation numbers we omitted year since first publication (*s*_*i*,*t*_) because the models had difficulty converging and the model fit was better without this variable. To test whether children had any impact we compared the above models with a null model with no child parameters:

$$\begin{array}{c} \text{Model}\;0:\;\text{log}\left(\mu_{i,t}\right)=\alpha_{0}+\alpha_{1}y_{i,t}+\alpha_{2}s_{i,t}+\gamma_{i} \end{array}$$

The models were compared using the Akaike Information Criterion (see below).

The collaboration outcomes used similar GLMM equations but the *t* index corresponded to publication rather than time. We used a binomial distribution with a logit link function for the outcomes of first author and last author. For the outcomes of first author, last author, and the number of countries outside Australia we also controlled for the total number of authors as this variable was a strong predictor of these outcomes.

#### Non-linear change over time

The change in the impact of caring for a child over time was modelled using the time-varying variable of time since child. We had no prior idea about the shape of this impact. For example, it could be non-linear with a stronger impact in the first few years. To allow for a range of shapes we used fractional polynomials which give interpretable non-linear curves [24]. To select the best model we used the Akaike Information Criterion (AIC) which is a trade-off of model fit and complexity [25]. The AICs were compared across the models using one or two children, and for the polynomial powers of: −2, −1, −0.5, 0, 0.5, 1, 2, 3, where for 0 we use the natural log transform. To help with model convergence we standardised time since child after applying the fractional polynomial transformation.

To visualise the model estimates we used the best fitting model and plotted the predicted number of publications and citations for: i) a woman who had no children, and ii) a woman who had two children, 2 years and 4 months apart. This gap between children was the average in our sample. We also tabulated the models’ mean estimates together with 95% confidence intervals. Residual plots were used to check for outliers and homoscedasticity.

#### Sample size

There are no readily available sample size calculators for our longitudinal study design, we therefore simulated data to estimate the required sample size. We randomly simulated paper numbers per year using a Poisson distribution. We included an increase over a woman’s career of 0.2 papers per year for all women based on past experience from a related study. To be able to detect a 0.15 decrease in publication counts for women caring for a child requires 110 women who have had a baby and 110 who have not. This gives 76% power and uses a 5% significance level. A 0.15 decrease represents a substantial slow down from the baseline annual increase of 0.2.

### Non-responders

To assess a potential non-response bias we compared the available publication data between women who did and did not complete the survey. For total citation numbers, paper numbers and first publication year, we used a two-sample t-test to compare responders and non-responders. For the number of authors and affiliations, and first or last author data, we used a generalised linear model with a random intercept for each woman to control for repeated data using the *lme4* library [26]. We used a Poisson distribution for the number of authors and affiliations and binomial distribution for the first and last author.

We also assessed the impact of non-response by using multiple imputation [27]. We had the complete citation and publication history for every woman, and therefore we only needed to impute their child caring data. Child data in three categories (none/1 child/2+ children) were randomly imputed using a multinomial distribution with probabilities based on the complete cases. For women randomly selected to have one or more children, the children’s birthdays were randomly generated based on the empirical data from the complete cases. Twenty multiply imputed data sets were analysed and combined using the *mitools* package [28].

### Sensitivity analyses

Sensitivity analyses were used to identify influential women. We searched for influential women by leaving out each woman in turn, re-running the analyses, and differencing these estimates from the estimates including all women, and then plotting the differences. This is the delta-beta statistic for identifying influential observations [23].

All analyses used R version 3.4.1 [29].

## Results

### Participants

Of the 465 women sampled, 160 women were eligible to participate and emailed the online survey. Ninety-five (59%) women fully completed the survey, 8 (5%) returned an incomplete survey that could not be included in the complete case analysis, and 65 (41%) did not respond. Of the responders, 59 had cared for children and 36 had not cared for any children (Table 1).

**Table 1:** Summary statistics on the women (n = 95) in terms of number of children, research output and research collaboration. The denominator for the per paper statistics is 3, 845 papers. IQR = inter-quartile range.

Number of children	n	%	Research output	Mean	Median	IQR
0	36	38	Publications per year	3.5	2	1–5
1	16	17	Citations per year	67	17	3–56
2	32	34	Research collaboration	Mean	Median	IQR
3+	11	11	Number of authors per paper	6.5	5	3–7
Author order on paper	n	%	Number of affiliations per paper	3.9	3	2–4
First author	1126	29	Number of affiliations outside Australia	0.4	0	0–0
Last author	904	24				

The average length of follow-up (or career length) for each woman was approximately 12 years and the total years of follow-up for the 95 participants was 1,135 years.

The differences between responders and non-responders in terms of publications and citations were small, although responders had a more recent first publication (Table 2). There were no clear differences between responders and non-responders in terms the number of authors or affiliations per paper. Responders had a somewhat reduced odds of being a last author, suggesting that our sample somewhat under-represents senior researchers.

**Table 2:** Comparisons of responders (*n* = 95) and non-responders (*n* = 65) for the publication and collaboration outcomes. The denominator for the per paper statistics is 3, 845 papers. The per paper mean difference/odds ratio, confidence intervals and p-values are from a mixed regression model with a random intercept per woman.

	Mean responders	Mean non-responders	Mean difference	95% CI for mean difference	p-value
First year of publication	2003.1	2000.6	–2.5	–5.1 to 0.3	0.08
Publications per year	3.1	3.3	0.2	–0.3 to 0.5	0.51
Citations per year	5.2	5.5	0.3	–0.3 to 0.9	0.27
Authors per paper	6.5	6.2	0.0	–0.1 to 0.2	0.86
Affiliations per paper	3.9	3.7	0.0	–0.1 to 0.2	0.56
	Percent for responders	Percent for non-responders	Odds ratio	95% CI for odds ratio	p-value
First author	29	33	0.94	0.69 to 1.28	0.69
Last author	24	24	0.69	0.47 to 1.00	0.05

### Yearly publications and citations

The best models for publications and citations both included terms for two children according to the AIC (Table A4 S2 Appendix).

**Fig 1:**
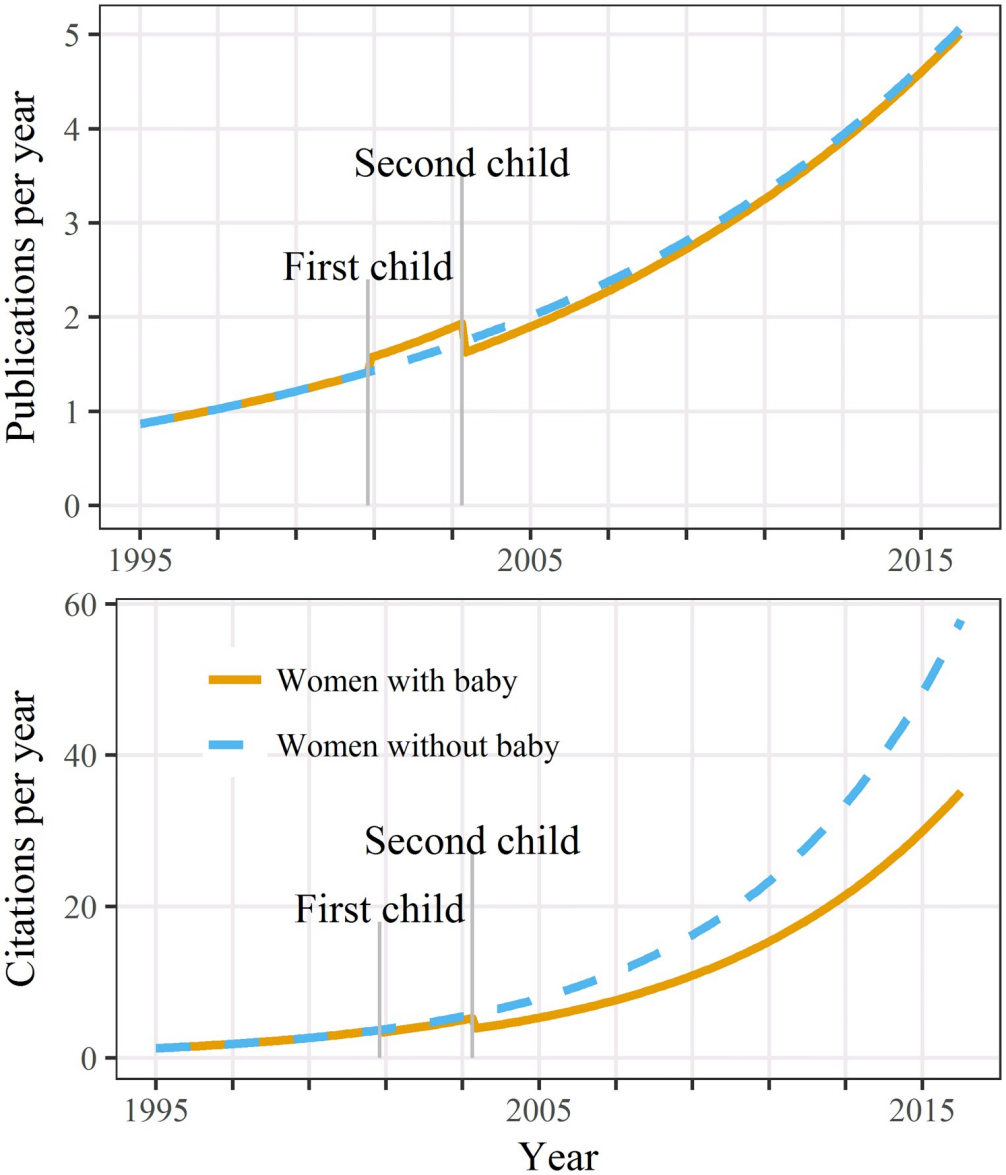
Estimated publications and citations over time for women caring for two children and women caring for none. Estimates shown for a career beginning in 1995.

There were relatively small associations between caring for children and publication counts as shown in Fig 1. There was a small increase in publication numbers after the first child (mean = 0.12), but this increase reversed after the second child (mean = −0.18; time-fixed predictors in Table 3). Both years since child predictors were strongly statistically significant (time-varying predictors in Table 3) although again the negative second child estimate (mean = –0.53) effectively cancelled the positive first child estimate (mean = 0.53).

**Table 3:** Estimated means and 95% confidence intervals from a longitudinal regression model with predictors for two children. Results on a log-scale for the numbers outcomes and log odds scale for the two author order outcomes. The best model for the probability of being last author only included the first child.

Outcome	Predictor	Mean	95% CI	p-value
Number of publications per year	First child, time-fixed	0.12	−0.05 to 0.28	0.17
	Second child, time-fixed	−0.53	−0.67 to −0.39	<0.001
	First child, time-fixed	−0.18	−0.35 to −0.01	0.037
	Second child, time-varying	0.53	0.38 to 0.69	<0.001
Number of citations per year	First child, time-fixed	−0.10	−0.15 to −0.04	<0.001
	Second child, time-fixed	−0.58	−0.64 to −0.51	<0.001
	First child, time-varying	0.39	0.32 to 0.47	<0.001
	Second child, time-varying	−0.31	−0.36 to −0.26	<0.001
Number of authors per paper	First child, time-fixed	0.28	0.20 to 0.36	<0.001
	Second child, time-fixed	−0.28	−0.35 to −0.20	<0.001
	First child, time-varying	0.03	−0.09 to 0.15	0.61
	Second child, time-varying	0.07	−0.03 to 0.17	0.16
Number of affiliations per paper	First child, time-fixed	0.01	−0.12 to 0.14	0.89
	Second child, time-fixed	−0.11	−0.24 to 0.01	0.07
	First child, time-varying	0.27	0.11 to 0.44	0.001
	Second child, time-varying	−0.21	−0.35 to −0.08	0.002
Number of countries outside Australia per paper	First child, time-fixed	−0.05	−0.37 to 0.27	0.76
	First child, time-varying	−0.26	−0.54 to 0.02	0.067
First author on paper	First child, time-fixed	0.29	−0.06 to 0.65	0.10
	Second child, time-fixed	−0.27	−0.63 to 0.08	0.13
	First child, time-varying	0.29	−0.08 to 0.66	0.13
	Second child, time-varying	−0.42	−0.78 to −0.06	0.022
Last author on paper	First child, time-fixed	−0.10	-0.54 to 0.34	0.66
	Second child, time-fixed	0.44	0.02 to 0.86	0.041
	First child, time-varying	−0.58	−0.98 to −0.17	0.005
	Second child, time-varying	0.37	−0.01 to 0.74	0.054

For citation counts, all four predictors were strongly statistically significant and three of the four had a negative impact on citation counts (Table 3). The largest impact is after a second child and the gap in citations widens over time (Fig 1).

### Collaboration outcomes

**Fig 2:**
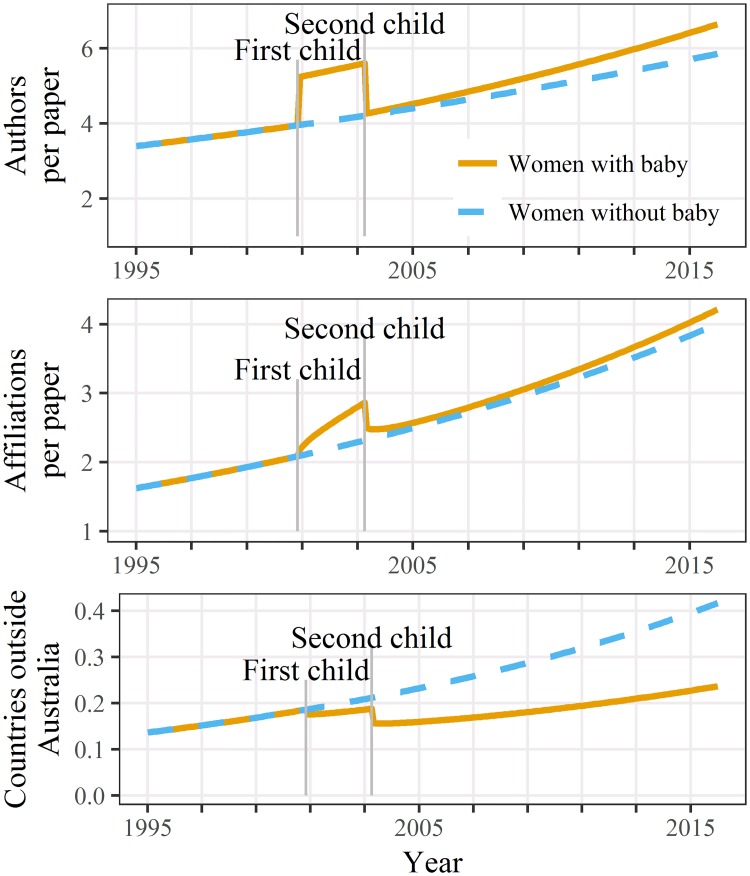
Estimated number of authors, affiliations and co-author countries outside Australia per paper over time for women caring for two children and women caring for none. Estimates shown for a career beginning in 1995.

The best model for the number of co-author countries outside Australia included just the first child, whereas the models for author and affiliation numbers both included two children (Table A4 S1 Appendix). The estimated changes over time are plotted in Fig 2. The estimated impact of caring for children was similar for the number of authors and affiliations, with an increase after the first child and decrease after the second. Caring for a child reduced the number of co-author countries outside Australia, although this decrease was not statistically significant (Table 3). However, there was less statistical power to detect a change in this variable given its relatively small mean (Table 1).

### Author order

**Fig 3:**
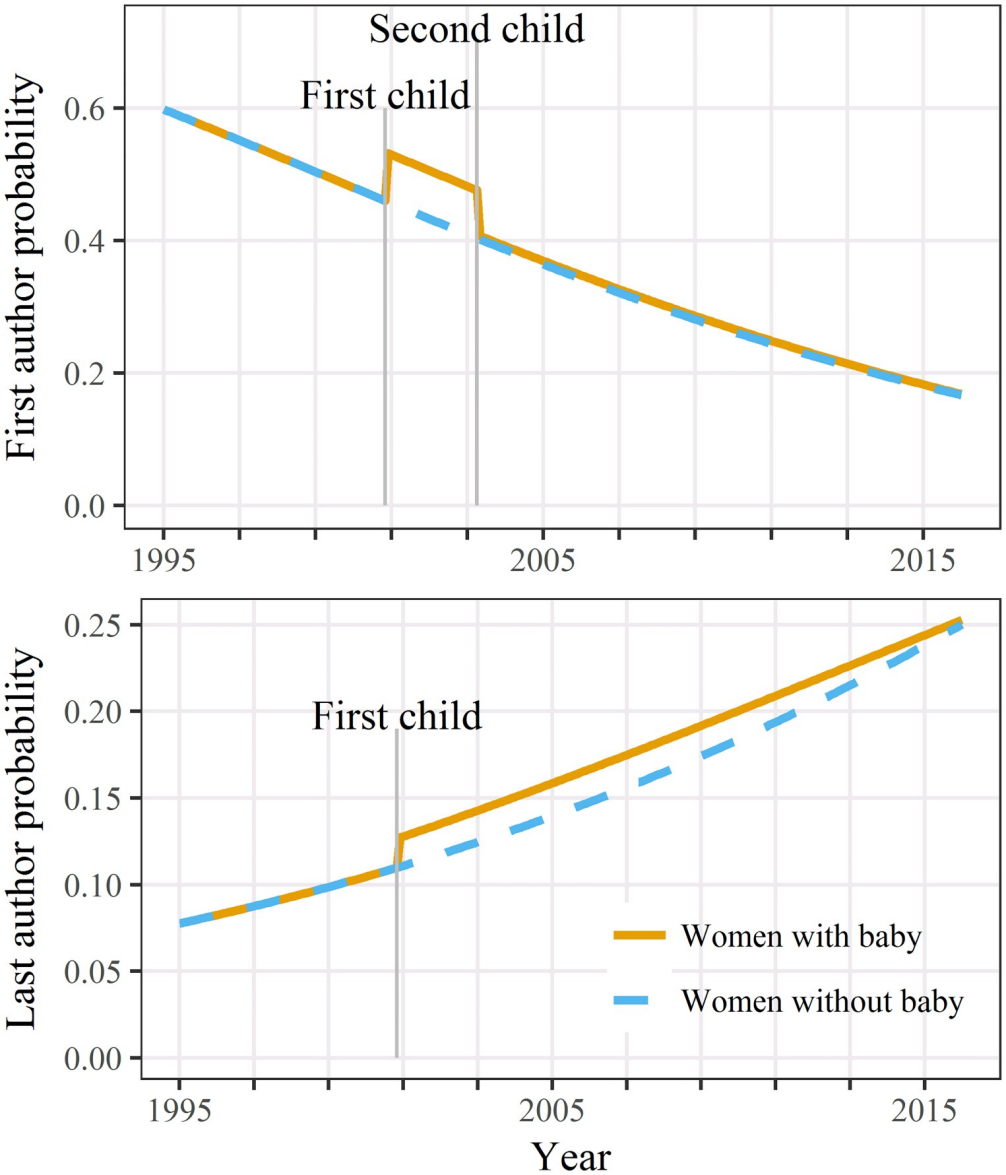
Estimated probability of being first or last author over time for women caring for two children and women caring for none. Estimates shown for a career beginning in 1995.

For both first and last author the best model used two children (Table A4 S1 Appendix). The probability of being the first author was slightly higher between the first and second child (Fig 3), although this change was not statistically significant (Table 3). The probability of being the last author was increased for women after their second child and this increase was statistically significant.

### Sensitivity analyses

The plots of the influential diagnostics (Figs A2 to A4 in S2 Appendix) showed there were statistically influential women for many of the outcomes. There were three key influential women (numbers 29, 43 and 57), and they all had children during their career and were in the top 10% of overall citation and publication counts.

**Fig 4:**
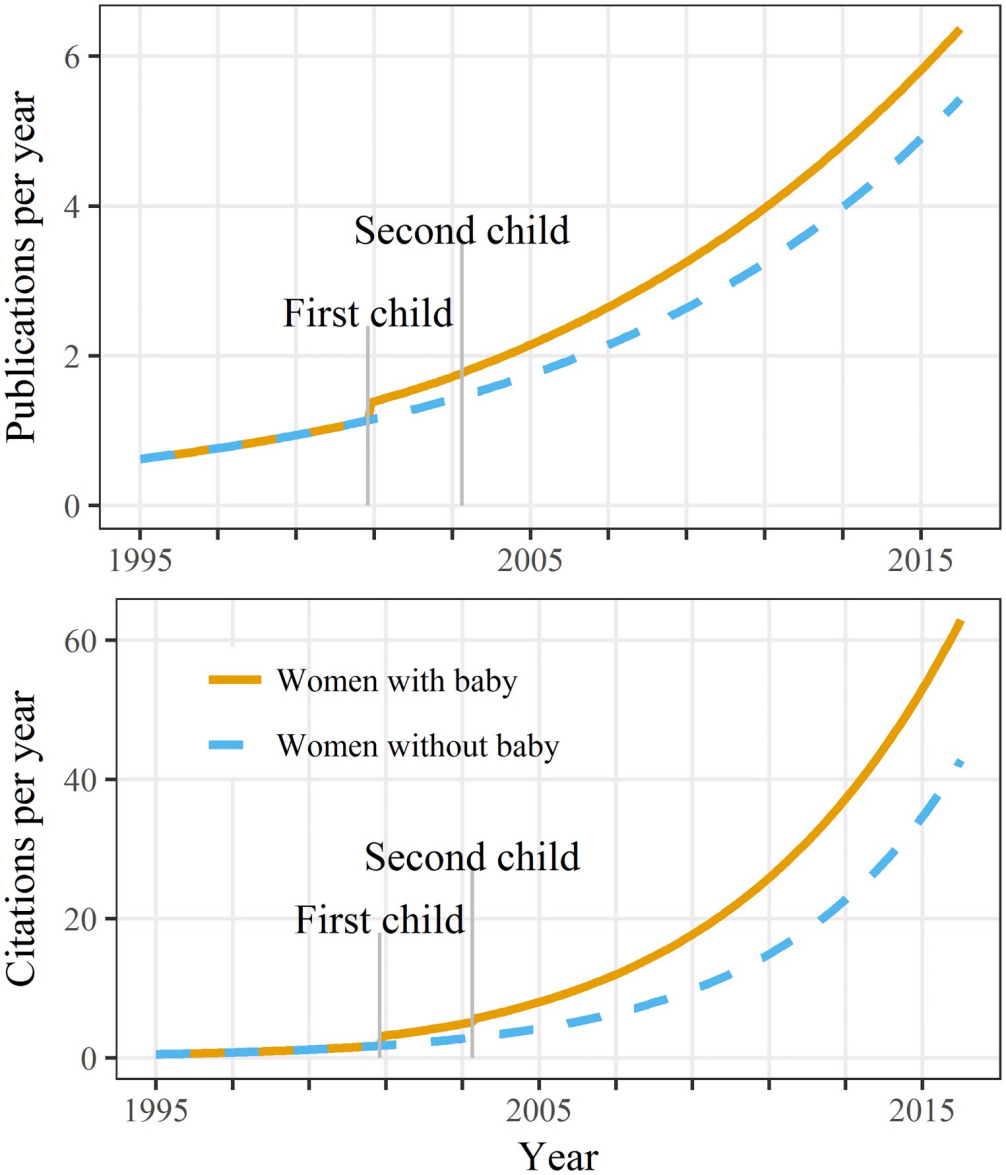
Estimated publications and citations over time for women caring for two children and women caring for none after leaving out one statistically influential woman. Estimates shown for a career beginning in 1995. Compare with Fig 1.

After re-running the publication model without the influential woman (number 29) there was a large change to the model’s estimates. The refitted model showed an increase in publication numbers after the first child that persisted over women’s careers (Fig 4). There was a smaller and not statistically significant increase in publication counts after the second child (Table A3 in S2 Appendix).

The refitted model for citation counts showed a similar pattern to publication counts (Fig 4), with an increase after the first child that persisted over women’s careers, and a smaller increase after the second child.

**Fig 5:**
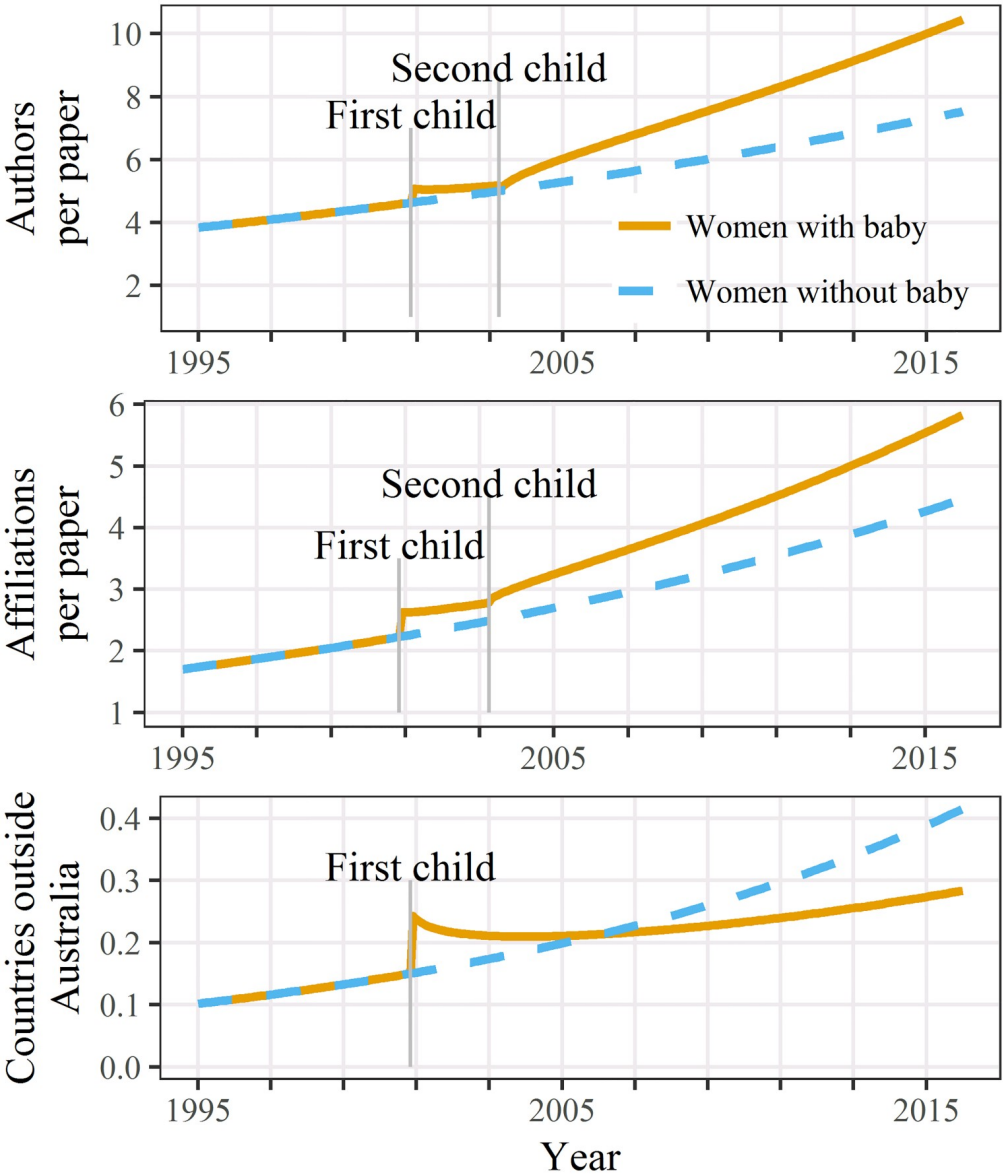
Estimated number of authors, affiliations, and co-author countries outside Australia per paper over time for women caring for two children and women caring for none after leaving out two statistically influential women. Estimates shown for a career beginning in 1995. Compare with Fig 2.

There were two influential women for the three networking outcomes (Fig A3 in S2 Appendix). After re-running the model without these women (numbers 29 and 57) the increase in author numbers after the first child was greatly reduced (Fig 5) although it remained statistically significant (Table A3 in S2 Appendix).

For the number of affiliations, the estimates over time without the two influential women showed a bigger increase in affiliations for women with children compared with those without (Fig 5 compared with Fig 2). For the number of countries outside Australia, there was now a rise in overseas co-authors which then remained flat and was overtaken by the estimated average for women without children.

For author order there was an influential woman on being last author and no influential women on being first author (Fig A4 in S2 Appendix). After removing the influential woman there was little change in the estimates for author numbers over time (Fig A5 in S2 Appendix).

### Multiple imputation

The multiple imputation results for citation and publication counts showed that missing data due to non-response may not have impacted the complete case mean estimates, although the estimates using imputed data had wider confidence intervals because of the uncertainty caused by the missing childcare histories (Fig A6 in S2 Appendix). For author and affiliation numbers per paper there was a change after imputing the childcare histories of women who did not respond (Fig A7 in S2 Appendix), however this had only a minor impact on the estimated numbers over time (Fig A8 in S2 Appendix). The multiple imputation for the author order outcomes showed no major change from the complete case analysis (Fig A8 in S2 Appendix).

## Discussion

Our objective was to determine if caring for children impacts on a woman’s output over her career. Our results revealed a complex picture, with differential impacts on output depending on the number of children, the outcome considered, and the presence of statistically influential women.

In the complete sample of women, caring for children did not greatly impact on a woman’s publication counts over her career (Fig 1). Women experienced a small increase in publications after the first child, followed by a decrease after the second child. Caring for children was negatively associated with citations, and this impact was greater for women who cared for two children. This result is particularly important given that the majority of respondents cared for two children (Table 1) and the most recent data from the Australian Bureau of Statistics showed that the total fertility rate in Australia is 1.8 [30].

Three women were highly statistically influential and the results were different when these women were excluded. We therefore should be cautious about generalising our results to the population of Australian female researchers. It may be an impossible task to provide a meaningful average impact and instead, as previous qualitative research indicated [14], the impact needs to be individually assessed.

Influential individuals are likely in such studies where there are a relatively small number of very active researchers meaning that publication and citation counts are positively skewed (Fig A4 in S2 Appendix). Previous studies may have also found influential researchers if they performed leave-one-out sensitivity analyses, and such influential researchers may explain the large differences in results between previous studies (see next section).

### Comparison with other studies

The impact of children on women’s output has been extensively studied since a seminal study by Cole and Zuckerman [5] which introduced the concept of “the productivity puzzle” [12]. Since this cornerstone study, many researchers have sought to explore this concept and determine if academic women are disadvantaged by caring for children.

Our finding that research output increased after caring for one child and decreased after caring for two children corroborated the findings of Hunter and Leahey [10]. Hunter and Leahey found that in the year after the birth of a child there was an immediate and significant increase in women’s output. They suggested this increase was the result of women increasing their work rate before having a child in anticipation of less time available in the future, in addition to publishing lag times. Hunter and Leahey’s study also showed that women with children experienced a decline in long-term output. Our findings partly support this notion, but only in the case of women caring for two children.

Few other studies have examined the relationship between number of children and research output. Also, when the age of children was taken into consideration, studies tended to evaluate the impact of young versus older children, or one versus many children. For example, Fox [31] found that women with preschool children were especially productive compared with women with school-aged children and/or women without children. Fox identified a number of potential reasons for this disparity, including: number of children (i.e., women with preschool children had fewer children overall), the personality traits of women (e.g., stamina and commitment to research), and time management (i.e., women with children dedicated their time to work and children only). Long [7] also examined the relationship between number of children and predoctoral publications. They found that mean publication counts for men and women declined with increasing numbers of children. However, this decline was much steeper for women. Long suggested this decrease in mean publishing output was the result of women’s reduced collaboration.

Some studies [6, 32] have provided evidence of a positive association between children and research output. Sax et al [6] found that having children did not impact on a woman’s research output. Their research also demonstrated that women devoted fewer hours to work when publishing at the same rate as men. For example, when they compared men and women with children who published 5 to 10 papers over a two-year period, they found that women with children allocated approximately 43 hours per week to research/writing and 38 hours to household/childcare. However, the men in this group devoted approximately 54 hours per week to research/writing and 26 hours to household/childcare. The idea that women with children use their time more efficiently is reflected in the following comment from our sample:

“My hours at work were so few, and the opportunity cost of being away from my kids so high, that I had to treat every working hour as something precious and scarce. As a result, I became much better at doing only work that really mattered to me and had a real potential to make a difference.”

(Participant 11 in S3 Appendix).

This idea is supported by the increase in last authorship probability after a second child (Fig 3) assuming this is a sign of the women focusing on research where they have greater involvement.

Some studies have found a negative association between children and research output. An Australian study by Bentley [33] examined the output of women with children. Their results provided weak evidence of an association between children and declines in publishing (i.e., article equivalents/year). However, as acknowledged, the results were limited by poorly defined variables. For example, Bentley did not specify the ages of children or the total number of children. They also observed an impact of child and elder care on output, but similarly, evidence of a negative impact on output was weak. Other shortcomings were acknowledged, including the fact that there was no consideration of when career disruptions occurred or the nature of the disruption, e.g. series of breaks or a single extended break.

The inclusion of elder care warrants further investigation. In our study, a number of women acknowledged the impact of caring for elders on output. For example, a participant stated,

“I appreciate that your study is focusing on children, however, have you considered that women are also often the primary carers for others, e.g. parents etc?”

(Participant 8 in S3 Appendix).

The impact of children on citations has not been as extensively studied as the impact on publications. However, it is gaining interest as institutes increasingly use citations to assess research quality. Hunter and Leahey [10] observed the citation counts of women with and without children. Their findings support our results that caring for children decreases women’s citation growth rate (i.e., the rate of increase of citations per year). They hypothesised that this was due to reductions in women’s networks and disciplinary alliances after the birth of a child, and/or the reduced likelihood of women submitting their work to prestigious journals.

An earlier study by Long [34] found that women experienced a decrease in citations from year four of their career, and that there was a larger gap in the median citation counts of men and women over their career.

Our results that caring for children negatively impacts on women’s citation counts supports further research into this area, particularly the factors contributing to these disparities. To date, a handful of studies have explored the reasons behind women’s poor citation rates. Kyvik and Teigen [35] found that women were less likely than men to collaborate with colleagues over a three-year period. The sample of women in this study indicated that ‘more internal collaboration on research’ and ‘more support and encouragement from colleagues’ might improve their research environment. They found that women with young children and women who did not collaborate were less productive. Figg et al [36] found a correlation between a woman’s level of collaboration (measured as a function of authors/article), and the number of times their work was cited. They concluded that the higher the level of collaboration, the greater the scholarly impact as indicated by citations per article. Geraci et al [37] suggested that women’s reduced citation rates were the result of: less professional networking by women due to family responsibilities (including attending conferences), greater service responsibilities, less institutional support, greater self-citation by males [38], and a tendency by males to cite works authored by other males.

The suggestion that reduced collaboration is the mediator between caring for children and reduced citation rates is supported by some women in our sample. A participant stated that,

“Child comes first always; this means missed opportunities for travel to research conferences.”

while another felt that,

“Caring for a child reduced one’s opportunity to participate fully in academic life and miss things like seminars taking place after 5pm and you could just go to as many conferences as you wished to attend.”

An impact on the ability to travel overseas is supported by the reduction in authors outside Australia after the first child (Fig 2).

### Limitations of this study

A key limitation of this study is the small sample size. However, we did collect detailed longitudinal data on each woman and had over 1,100 years of follow up. We also had a large enough sample size to show statistically significant differences for six of the seven outcomes (Table 3).

We did not meet our target sample size of 220 women and had a moderate response rate of 59%. Women who did not complete the survey could be an influential group, particularly if they ended their research career due to child-caring responsibilities. Excluding this group may bias the results and overstate the output of women with children. While efforts were made to contact these women (through email and mail) their response rate remained low. However, we were able to access the careers of all women and found no statistically significant differences between women who did and did not respond in terms of citations and papers (Table 2), which reduces any potential bias.

Entry into our sample was conditional on producing a first author paper between 2007 and 2015, meaning we missed women who were inactive during this period or who left research before 2007. We used this approach because we needed to find women who were research active in order to examine the change in their activity. Other studies in this field similarly condition on current activity and previous studies have used cross-sectional surveys [31], researchers who applied for grant applications [39], and academics on tenure track [10], which are all biased towards capturing more active researchers. By conditioning on first authors we likely included researchers who contributed more to that paper [40].

We used *Scopus* for the publication data and this system uses an algorithm to assign published papers to a unique scholar ID. This algorithm will not always correctly assign papers to researchers, particularly if a researcher changes their name after marriage. Researchers can send a request to *Scopus* to merge their records where two or more have been created. Two studies of the *Scopus* algorithm estimated the precision was of 87% and 99%, and the recall was 96% and 98% [41, 42].

We did not examine men as we expected the largest impact of child caring to be in women.

Future studies would ideally be: larger, include inactive researchers, and include male researchers in order to allow a comparison of the impact of caring for children by gender. More detailed data on career disruption would be useful, including carer’s responsibilities and detail on the career disruption in terms of the reduced hours and length of time to return to work, and whether the researcher returned to a different role.

### Policy implications

Many promotion committees and funding agencies aim to assess a researcher’s performance whilst adjusting for any career disruptions. However, the interpretation of disruption is left to peers and there are often no formal mechanisms for recognising the impact of caring for children, such as separate funding pools or increases to scores. Perhaps due to this, track record remains a dominant criterion for making funding decisions. In Mow [1] an interviewee stated that the heavy weighting given to track record was “because it was measurable and comparable and manageable” [1]. An interviewee in Mow’s study also stated the Australian Research Council uses track record because it is the “cheap way to do it” [1].

While it is promising that potential career disruptions such as pregnancy and carers’ responsibilities are now a mandatory component of funding applications, it is unclear how these interruptions are assessed and how they affect the outcomes of peer review processes. An attempt to establish a numerical formula for assessing an individual’s merit over their career has been made by Monash University’s Equal Opportunity for Women Committee [43]. Although our results suggest that one generalisable equation may be impossible.

There is emerging evidence that women who care for children collaborate less with their colleagues. This could be because of commitments outside of work, including greater household and child-caring responsibilities. These factors also need to be considered when assessing research output for the purposes of granting funding. It is not enough to view research performance only in the context of reduced work hours. These factors are also likely to affect women caring for children differently. For example, a single parent with limited family support may find it more difficult to attend networking events and collaborate with peers compared with women who have access to childcare and the support of a partner.

## Conclusions

The evidence from our study and previous literature is that—for some women—research output decreases after caring for a child and this may be due to reduced networking. Institutions concerned about this may want to offer greater support for female researchers with children, such as additional money to cover childcare during conferences. Institutions that use publication and citation benchmarks as a key criteria for appointment and promotion [44] should investigate ways to adjust the benchmarks for women who have cared for children.

## Supporting information

S1 Fig Flow diagram of participant recruitment. (PDF)

S1 Appendix Participant information sheet and online survey. (PDF)

S2 Appendix Additional tables and figures. (DOCX)

S3 Appendix Participants’ comments. (DOCX)
