# Supplementary figures and images for: The impact of caring for children on women’s research output: A retrospective cohort study
Source: PLoS One. 2019 Mar 21;14(3):e0214047. doi: 10.1371/journal.pone.0214047 (PMC6428253; doi:10.1371/journal.pone.0214047)

**S1 Figure – flow diagram of participants**

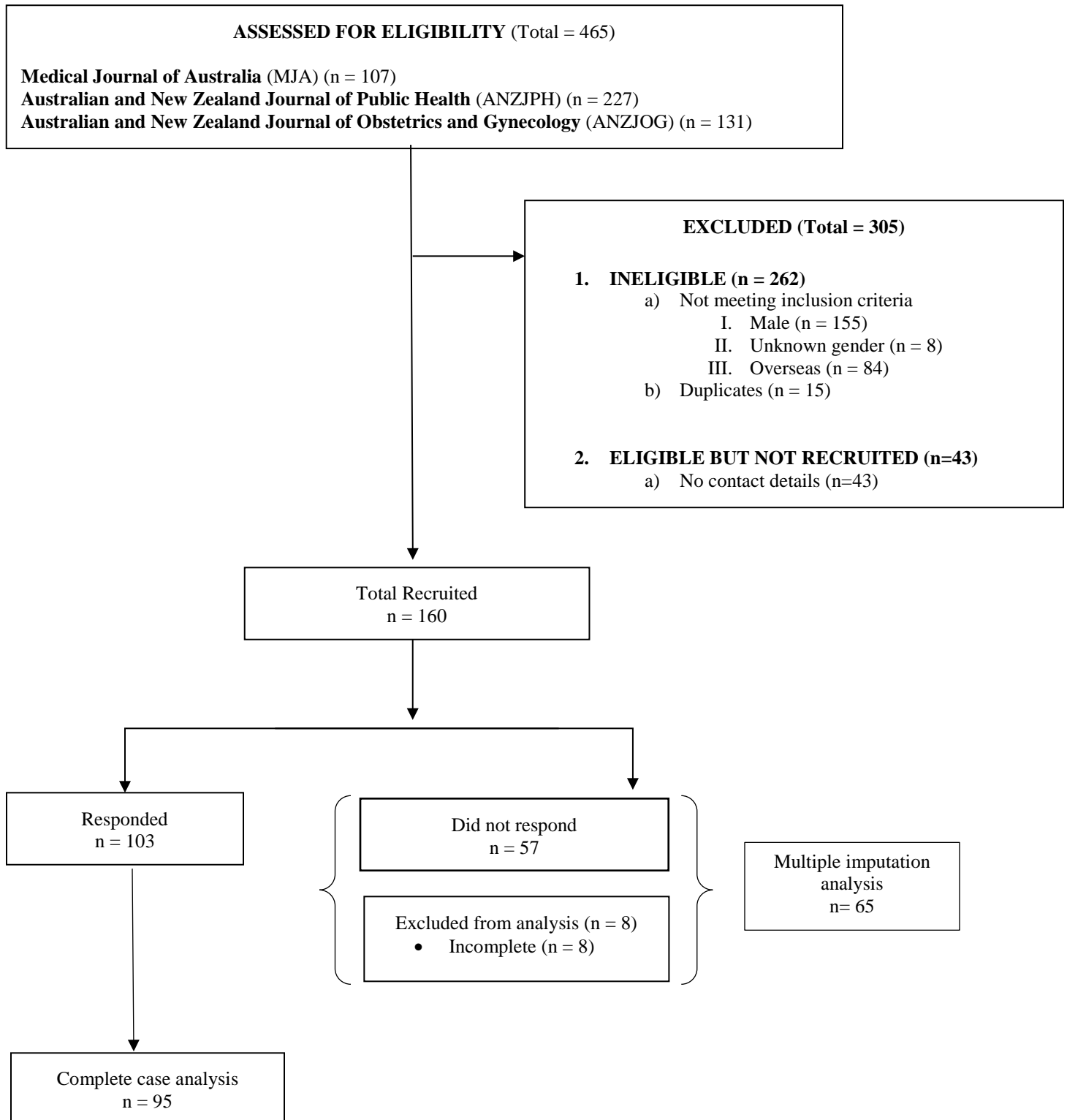

Supplement: S1 Fig — (PDF) [file pone.0214047.s001.pdf]
