# Supplementary material for: The impact of caring for children on women’s research output: A retrospective cohort study
Source: PLoS One. 2019 Mar 21;14(3):e0214047. doi: 10.1371/journal.pone.0214047 (PMC6428253; doi:10.1371/journal.pone.0214047)
Supplement: S1 Appendix — (PDF) [file pone.0214047.s002.pdf]

Impact of caring for children on women's research productivity

PARTICIPANT INFORMATION FOR QUT RESEARCH PROJECT The impact of caring for children on women's research productivity QUT Ethics Approval Number 1500001010 RESEARCH TEAMPrincipal Researcher:Adrian Barnett Associate ProfessorAssociate Researcher:Lauren Healey Research Asssistant School of Public Health Social Work Faculty of Health Queensland University of Technology (QUT)DESCRIPTIONThis project is being undertaken as part of our group's research into the impact of caring for children on women's research productivity. The purpose of this project is to quantify the impact of caring for children on women's research productivity by evaluating women's research output (i.e. number of publications), journal impact factor, and number of citations using data from Scopus. You have been invited to participate because a paper you published was randomly selected. PARTICIPATIONParticipation will involve completing a short online survey that will take approximately 5 to 10 minutes of your time. Your participation in this project is entirely voluntary. If you agree to participate you do not have to complete any question(s) you are uncomfortable answering. Your decision to participate or not participate will in no way impact upon your current or future relationship with QUT. If you do agree to participate you can withdraw from the project without comment or penalty. Please let a member of the research team know if you wish to withdraw from the study. You should be aware that data collected up to the time that you withdraw will form part of the research project results. If you do not want your data to be included, you must inform the researchers when you withdraw from the research project. EXPECTED BENEFITSIt is expected that this project will not directly benefit you. However, your data will contribute to providing the first quantitative evidence as to what extent women's research productivity is impacted by caring for a baby. The results could be used to inform workplace policies for making fairer comparisons between researchers for important decisions such as funding and promotion. RISKSThere are no risks beyond normal day-to-day living associated with your participation in this project. PRIVACY & CONFIDENTIALITYAll comments and responses will be treated confidentially unless required by law. Any data collected as part of this project will be stored securely as per QUT's Management of research data policy. Please note that non-identifiable data collected in this project may be used as comparative data in future projects or stored on an open access database for secondary analysis. CONSENT TO PARTICIPATESubmitting the completed online survey is accepted as an indication of your consent to participate in this project. QUESTIONS / FURTHER INFORMATION ABOUT THE PROJECTIf you have any questions or require further information please contact one of the researchers listed below. Adrian Barnett Lauren Healey Associate ProfessorResearch Assistant07 3138 601007 3138 6010a.barnett@qut.edu.aulauren.healey@qut.edu.auCONCERNS / COMPLAINTS REGARDING THE CONDUCT OF THE PROJECTQUT is committed to research integrity and the ethical conduct of research projects. However, if you do have any concerns or complaints about the ethical conduct of the project you may contact the QUT Research Ethics Advisory Team on 07 3138 5123 or email ethicscontact@qut.edu.au. The QUT Research Ethics Advisory Team is not connected with the research project and can facilitate a resolution to your concern in an impartial manner. Thank you for helping with this research project. Please keep this sheet for your information. By agreeing to participate in this study, you are agreeing that you: have read and understood the information provided in the Information to Participants sectionhave had any questions answered to your satisfactionagree to participate in this online surveyunderstand that once you have submitted your responses, these cannot be withdrawn

Please indicate your response to the statements above and consent to participate.

☐ I have read and understand the Participant Information Sheet and consent to participate in this study.

Have you ever cared for a child, either as a biological, adoptive, step-, or foster mother, and/or legal guardian?

○ Yes

○ No

How many children have you cared for?

- ○ 1
- ○ 2
- ○ 3
- ○ 4
- ○ 5
- ○ 6
- ○ 7
- ○ 8
- ○ 9
- ○ 10

	Date care commenced (MMM/YYYY) e.g. Aug/2000	Type of care	Time to return to paid work (casual, part-time, or full-time) in months
Child 1	....	○ Biological/adoptive parent<br>○ Step parent<br>○ Foster parent<br>○ Legal guardian/other	....
Child 2	....	○ Biological/adoptive parent<br>○ Step parent<br>○ Foster parent<br>○ Legal guardian/other	....
Child 3	....	○ Biological/adoptive parent<br>○ Step parent<br>○ Foster parent<br>○ Legal guardian/other	....
Child 4	....	○ Biological/adoptive parent<br>○ Step parent<br>○ Foster parent<br>○ Legal guardian/other	....
Child 5	....	○ Biological/adoptive parent<br>○ Step parent<br>○ Foster parent<br>○ Legal guardian/other	....
Child 6	....	○ Biological/adoptive parent<br>○ Step parent<br>○ Foster parent<br>○ Legal guardian/other	....
Child 7	....	○ Biological/adoptive parent<br>○ Step parent<br>○ Foster parent<br>○ Legal guardian/other	....
Child 8	....	○ Biological/adoptive parent<br>○ Step parent<br>○ Foster parent<br>○ Legal guardian/other	....
Child 9	....	○ Biological/adoptive parent<br>○ Step parent<br>○ Foster parent<br>○ Legal guardian/other	....
Child 10	....	○ Biological/adoptive parent<br>○ Step parent<br>○ Foster parent<br>○ Legal guardian/other	....

Thank you for completing this survey. We welcome any comments about this research or your experience of caring for children whilst working.

....

....

....

....
