# Supplementary material for: The impact of caring for children on women’s research output: A retrospective cohort study
Source: PLoS One. 2019 Mar 21;14(3):e0214047. doi: 10.1371/journal.pone.0214047 (PMC6428253; doi:10.1371/journal.pone.0214047)
Supplement: S2 Appendix — (DOCX) [file pone.0214047.s003.docx]

**Appendix S2 – Additional figures and tables**

**Table A1: Example longitudinal data structure (using hypothetical data)**

| Woman | Year | First child | Years since first child | Second child | Years since second child | Publications | Citations |
| --- | --- | --- | --- | --- | --- | --- | --- |
| 1 | 1995 | 0 | 0 | 0 | 0 | 1 | 0 |
| 1 | 1996 | 0 | 0 | 0 | 0 | 0 | 1 |
| 1 | 1997 | 0 | 0 | 0 | 0 | 0 | 4 |
| 1 | 1998 | 0 | 0 | 0 | 0 | 0 | 0 |
| 1 | 1999 | 0 | 0 | 0 | 0 | 5 | 3 |
| 1 | 2000 | 0 | 0 | 0 | 0 | 5 | 4 |
| 1 | 2001 | 0 | 0 | 0 | 0 | 3 | 12 |
| 1 | 2002 | 0 | 0 | 0 | 0 | 2 | 12 |
| 1 | 2003 | 0 | 0 | 0 | 0 | 2 | 17 |
| 1 | 2004 | 1 | 0.083333 | 0 | 0 | 6 | 19 |
| 1 | 2005 | 1 | 1.083333 | 0 | 0 | 9 | 12 |
| 1 | 2006 | 1 | 2.083333 | 0 | 0 | 2 | 24 |
| 1 | 2007 | 1 | 3.083333 | 1 | 0.75 | 1 | 22 |
| 1 | 2008 | 1 | 4.083333 | 1 | 1.75 | 4 | 33 |
| 1 | 2009 | 1 | 5.083333 | 1 | 2.75 | 4 | 14 |
| 1 | 2010 | 1 | 6.083333 | 1 | 3.75 | 2 | 33 |
| 1 | 2011 | 1 | 7.083333 | 1 | 4.75 | 2 | 35 |
| 1 | 2012 | 1 | 8.083333 | 1 | 5.75 | 5 | 55 |
| 1 | 2013 | 1 | 9.083333 | 1 | 6.75 | 7 | 43 |
| 1 | 2014 | 1 | 10.08333 | 1 | 7.75 | 8 | 33 |
| 1 | 2015 | 1 | 11.08333 | 1 | 8.75 | 5 | 44 |

**Table A2: Four predictors used to model the impact of caring for children on publication and citation counts over time**

| **Predictor** | **Description** |
| --- | --- |
| Caring for one child | Fixed change after first child |
| Caring for two children | Fixed change after second child |
| Years since care began for first child | Time-varying change after first child |
| Years since care began for second child | Time-varying change after second child |

**Figure A1: Example plot of model structure showing time and the four predictors in Table A2. In this example years since caring for first and second child have a linear effect, but this could be non-linear using the fractional polynomial approach.**


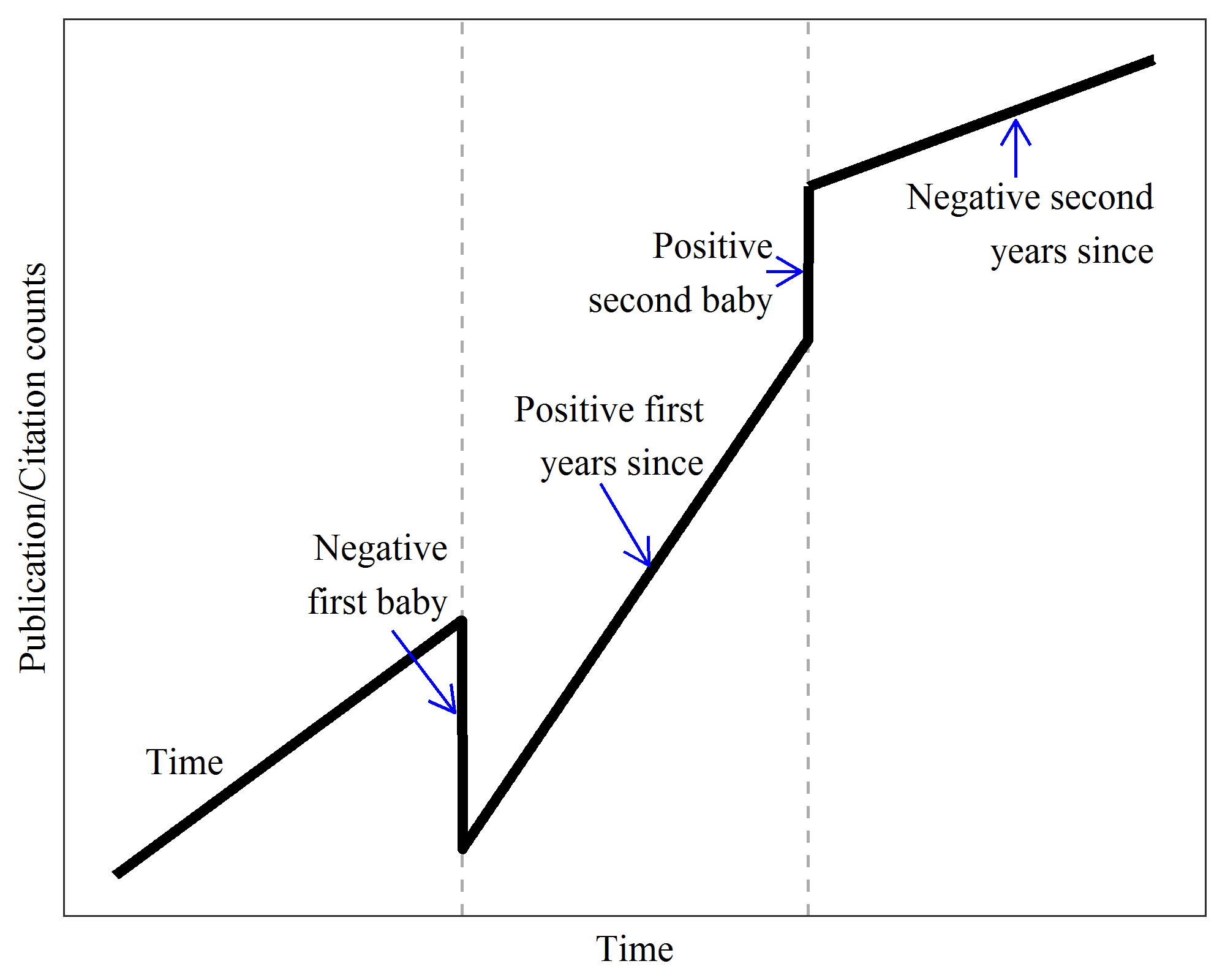


**Figure A2: Influential diagnostics for models of citations and papers using two children.** The blue dot shows the estimates without participant 29 who is influential on citations counts and to a lesser degree paper counts.


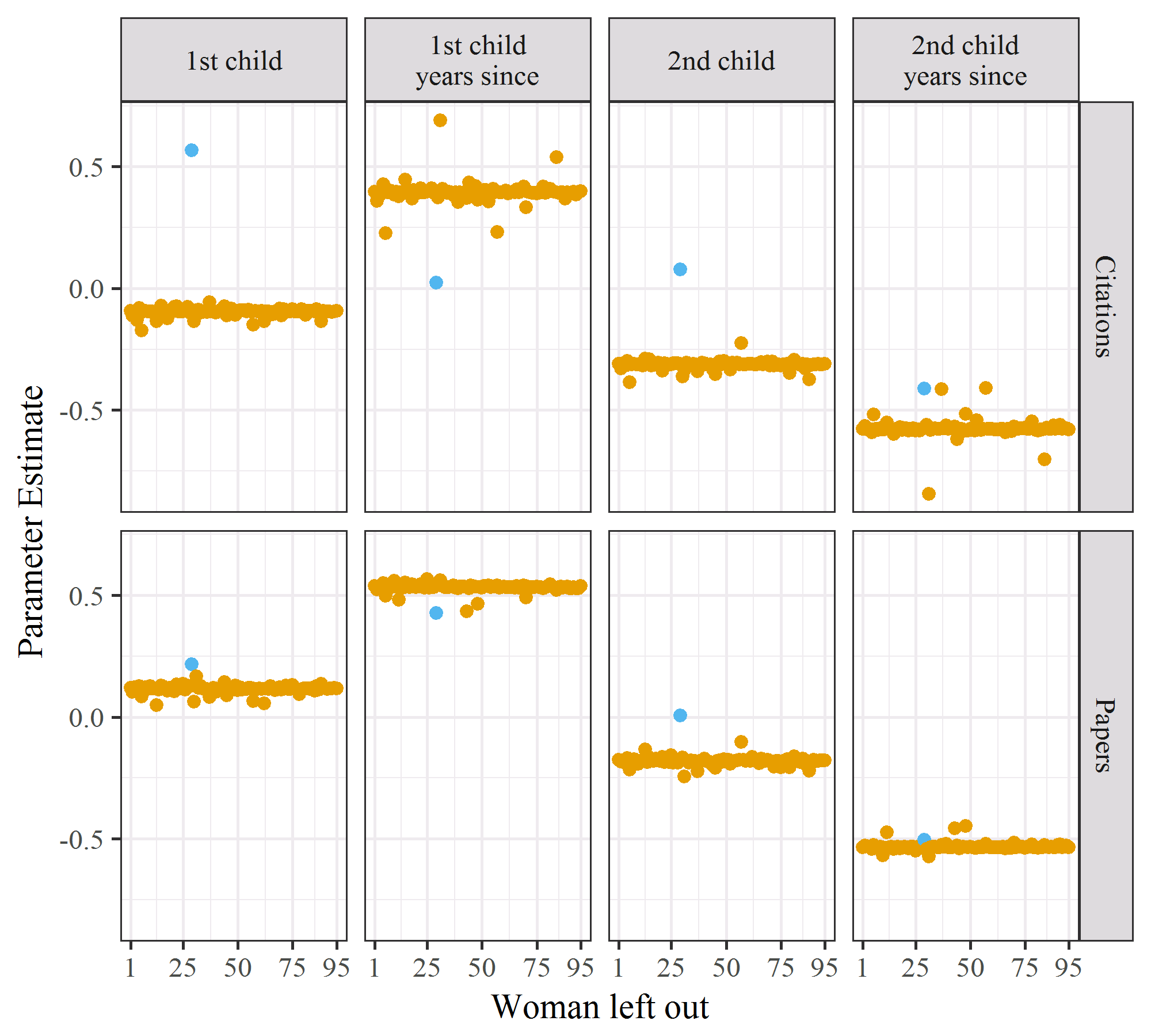


**Figure A3: Influential diagnostics for models of numbers of authors, affiliations and countries using two children.** The blue dot shows the estimates without participant 29 and the green dot participant 57 who are influential on all three outcomes.

**
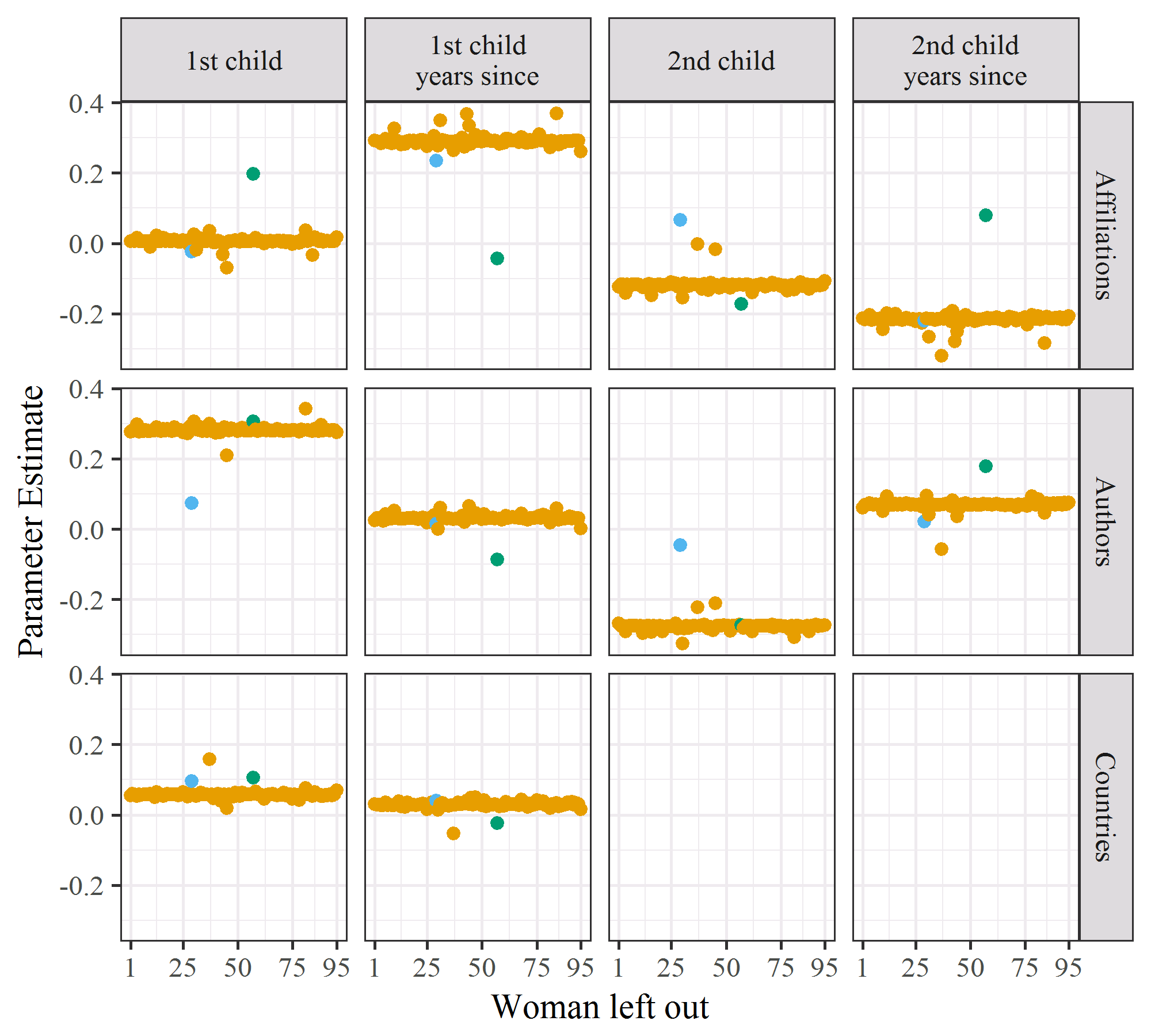
**

**Figure A4: Influential diagnostics for models of first and last author using two children.** The blue dot shows the estimates without participant 43 who is influential on the probability of being first author. The model for last author only included one child.

**
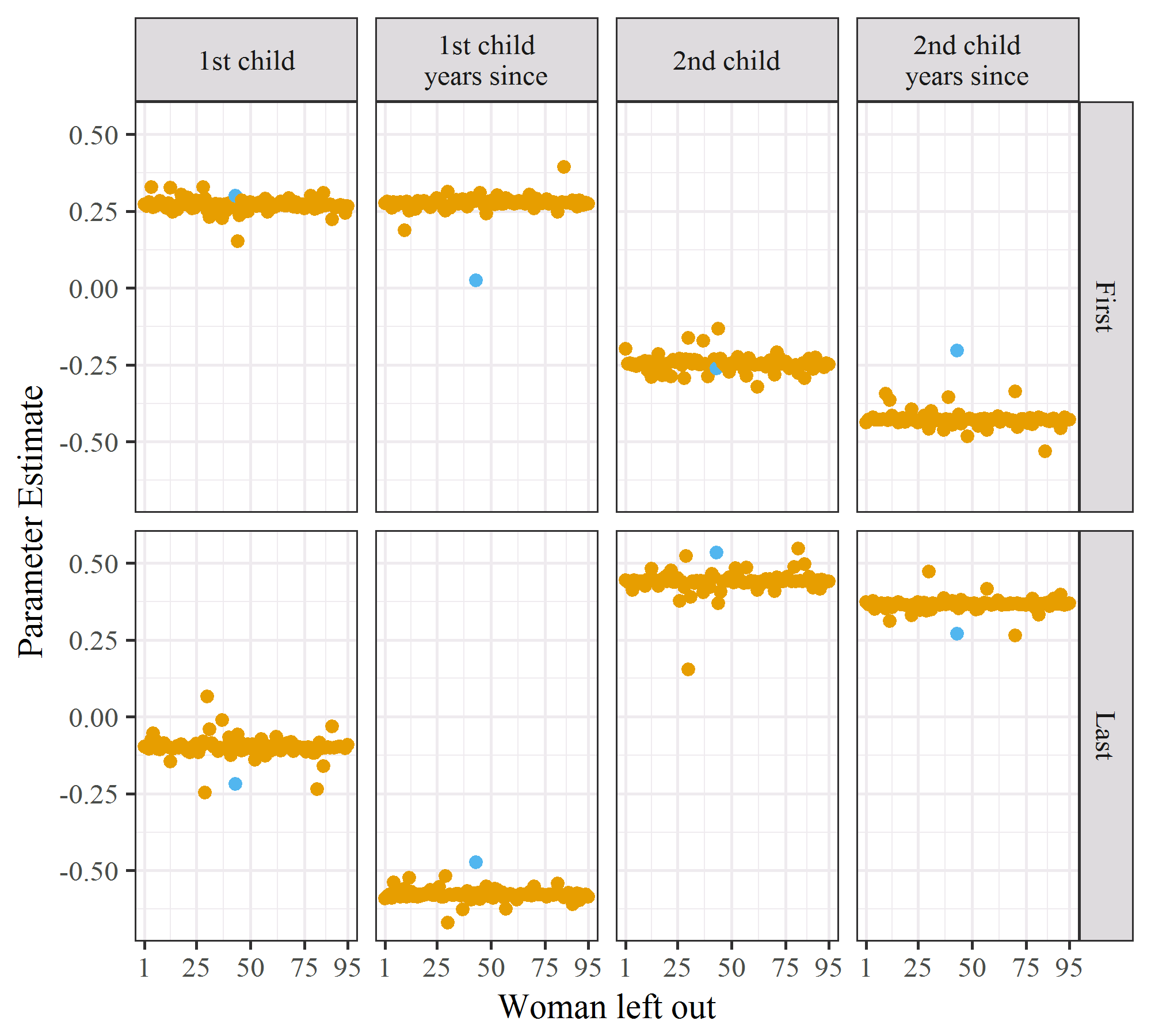
**

**Figure A5: Estimated probability of being last author for women caring for two children and women caring for none after leaving out one statistically influential woman.** Estimates for women whose careers started in 1995. Compare with Fig 3.

**
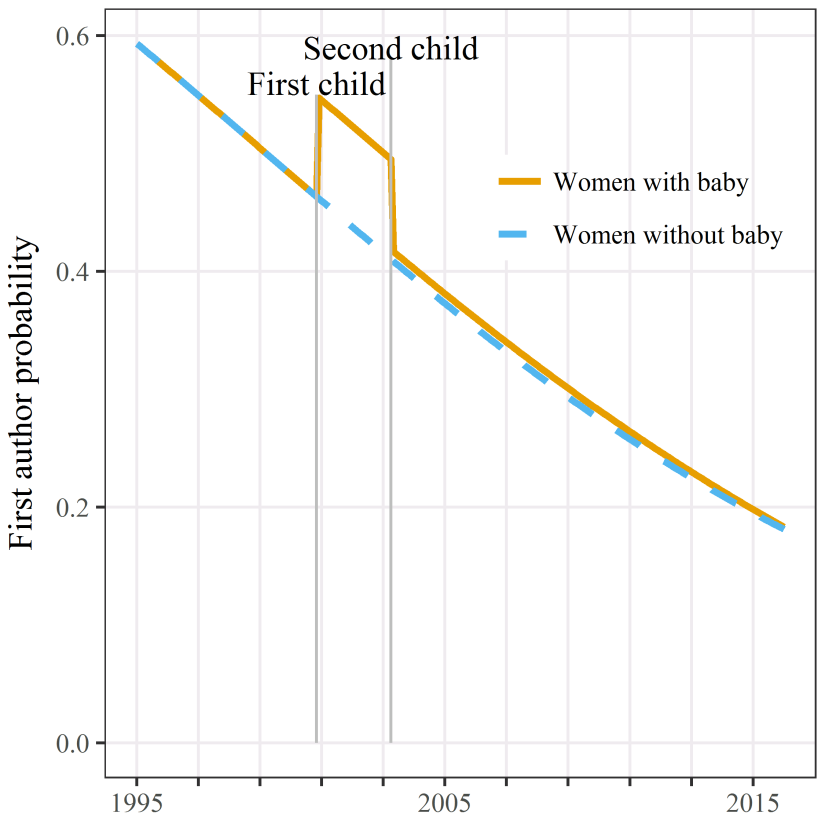
**

**Table A3: Estimated model parameters (log or logit scale) for women caring for two children after removing statistically influential women.**

| **Outcome** | **Predictor** | **Mean** | **95% CI** | **P-value** |
| --- | --- | --- | --- | --- |
| Publications | First child, time-fixed | 0.18 | 0.00 to 0.37 | 0.053 |
| (log scale) | Second child, time-fixed | 0.00 | –0.18 to 0.19 | 0.96 |
|  | First child, time-varying | 0.41 | 0.24 to 0.59 | <0.001 |
|  | Second child, time-varying | –0.51 | –0.66 to –0.36 | <0.001 |
| Citations | First child, time-fixed | 0.57 | 0.49 to 0.64 | <0.001 |
| (log scale) | Second child, time-fixed | 0.08 | 0.02 to 0.14 | 0.015 |
|  | First child, time-varying | 0.02 | –0.05 to 0.1 | 0.55 |
|  | Second child, time-varying | –0.41 | –0.47 to –0.34 | <0.001 |
| Authors | First child, time-fixed | 0.10 | 0.01 to 0.19 | 0.026 |
| (log scale) | Second child, time-fixed | –0.04 | –0.13 to 0.04 | 0.30 |
|  | First child, time-varying | –0.09 | –0.22 to 0.03 | 0.14 |
|  | Second child, time-varying | 0.12 | 0.02 to 0.23 | 0.019 |
| Affiliations | First child, time-fixed | 0.17 | 0.02 to 0.32 | 0.025 |
| (log scale) | Second child, time-fixed | 0.00 | –0.13 to 0.14 | 0.97 |
|  | First child, time-varying | –0.08 | –0.24 to 0.07 | 0.28 |
|  | Second child, time-varying | 0.07 | –0.07 to 0.20 | 0.33 |
| Countries | First child, time-fixed | 0.54 | 0.13 to 0.95 | 0.010 |
| (log scale) | First child, time-varying | –0.52 | –0.85 to –0.18 | 0.002 |
| First author | First child, time-fixed | 0.31 | –0.05 to 0.67 | 0.09 |
| (logit scale) | Second child, time-fixed | –0.29 | –0.65 to 0.07 | 0.12 |
|  | First child, time-varying | –0.01 | –0.62 to 0.61 | 0.96 |
|  | Second child, time-varying | –0.16 | –0.75 to 0.44 | 0.61 |

**Figure A6: Parameter estimates and 95% confidence intervals (log-scale) for citation and paper numbers for complete case and multiply imputed data.** The wider confidence intervals for the imputed data reflect the uncertainty caused by the missing child care data.


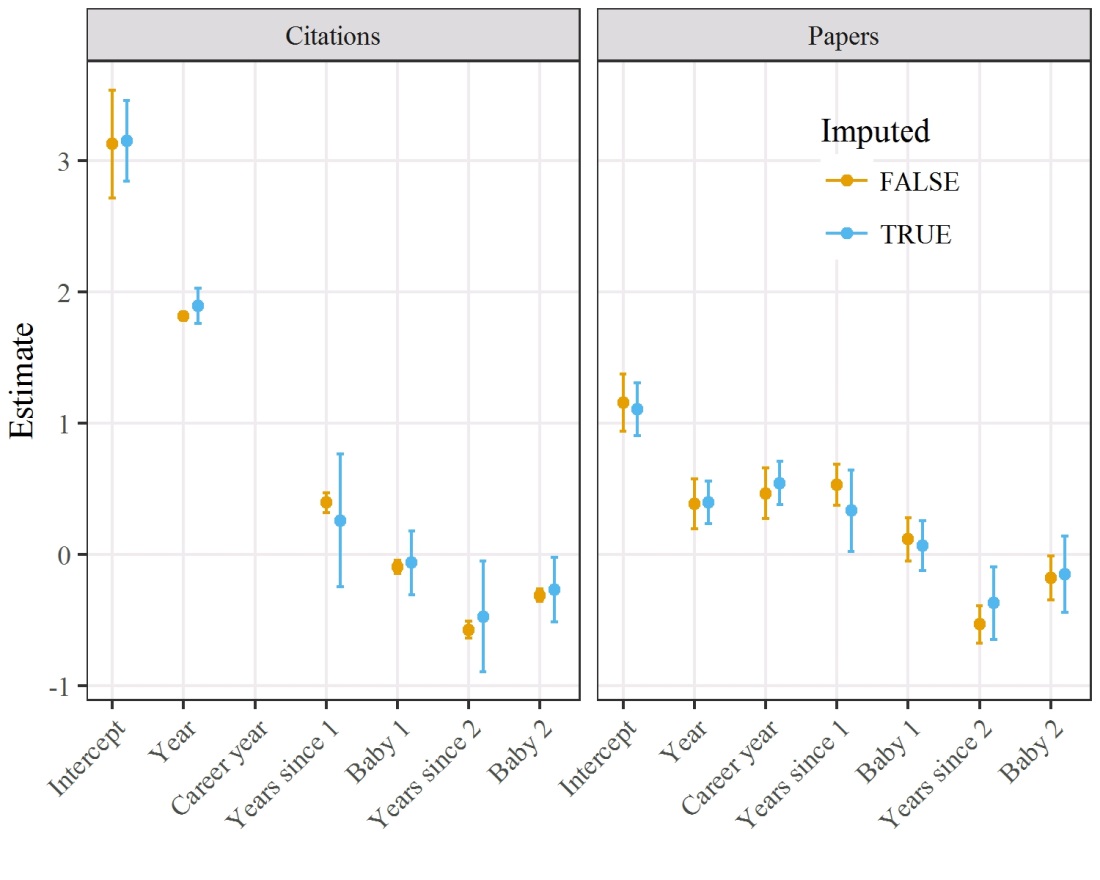


**Figure A7: Parameter estimates and 95% confidence intervals for author, affiliation and country numbers (log-scale) for complete case and multiply imputed data.** The wider confidence intervals for the imputed data reflect the uncertainty caused by the missing child care data. The imputed results for affiliation numbers are generally closer to the null.


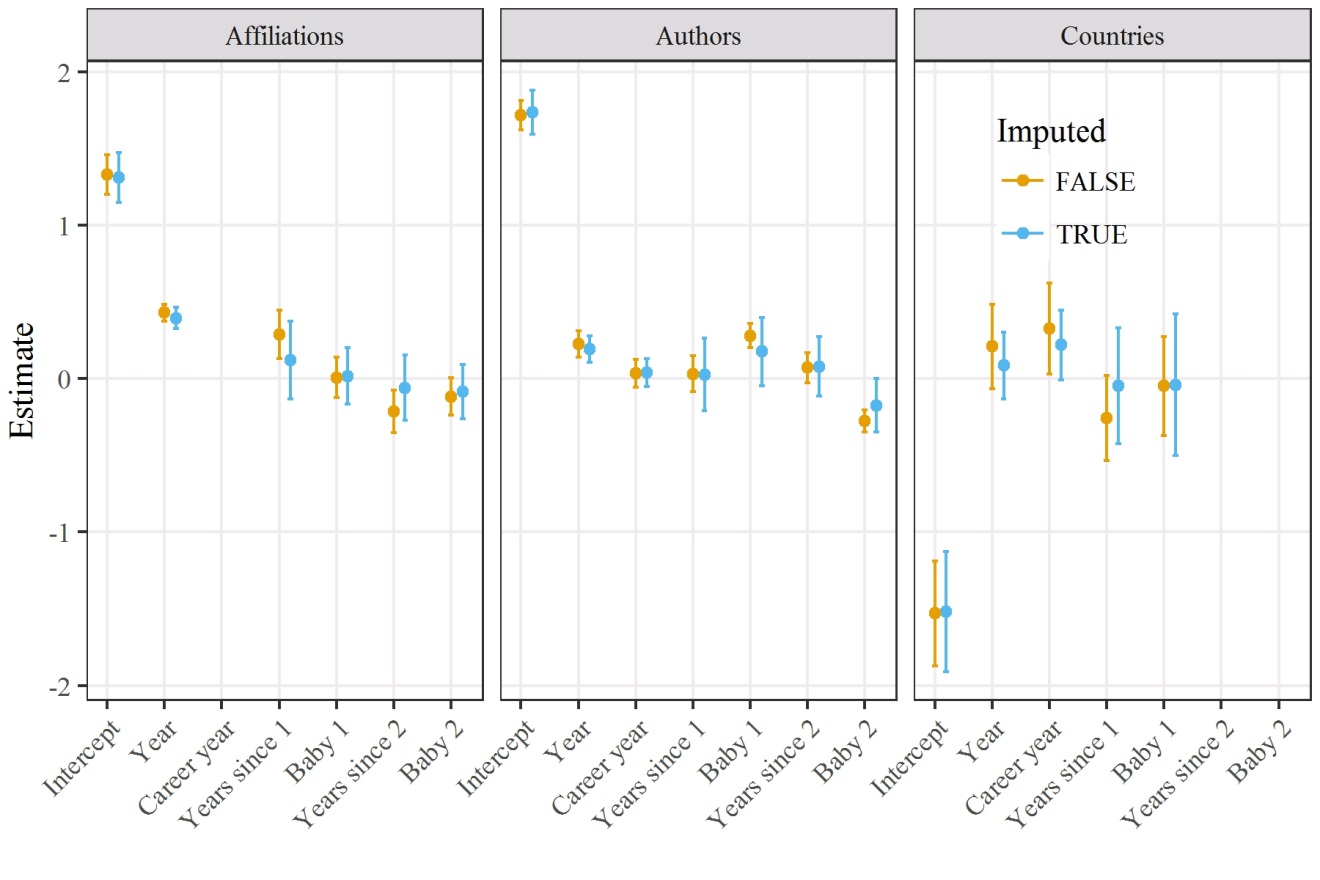


**Figure A8: Parameter estimates and 95% confidence intervals for probability of being first or last author (logit-scale) for complete case and multiply imputed data.**

**
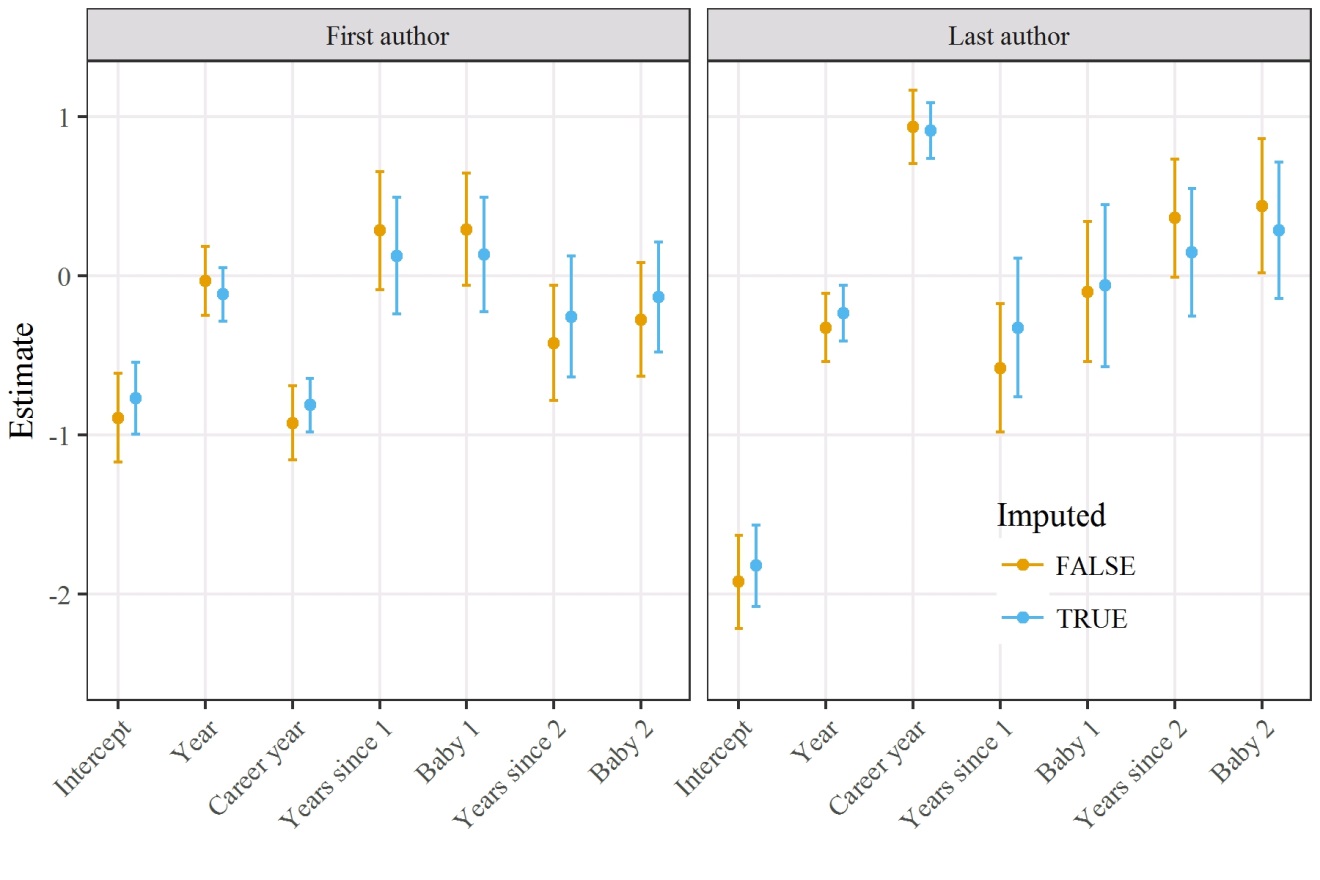
**

**Figure A9: Estimated number of authors, affiliations and countries outside Australia per paper over time for women caring for two children and women caring for none using estimates that impute non-responders.** Estimates for women whose careers started in 1995. Compare with Fig 2.


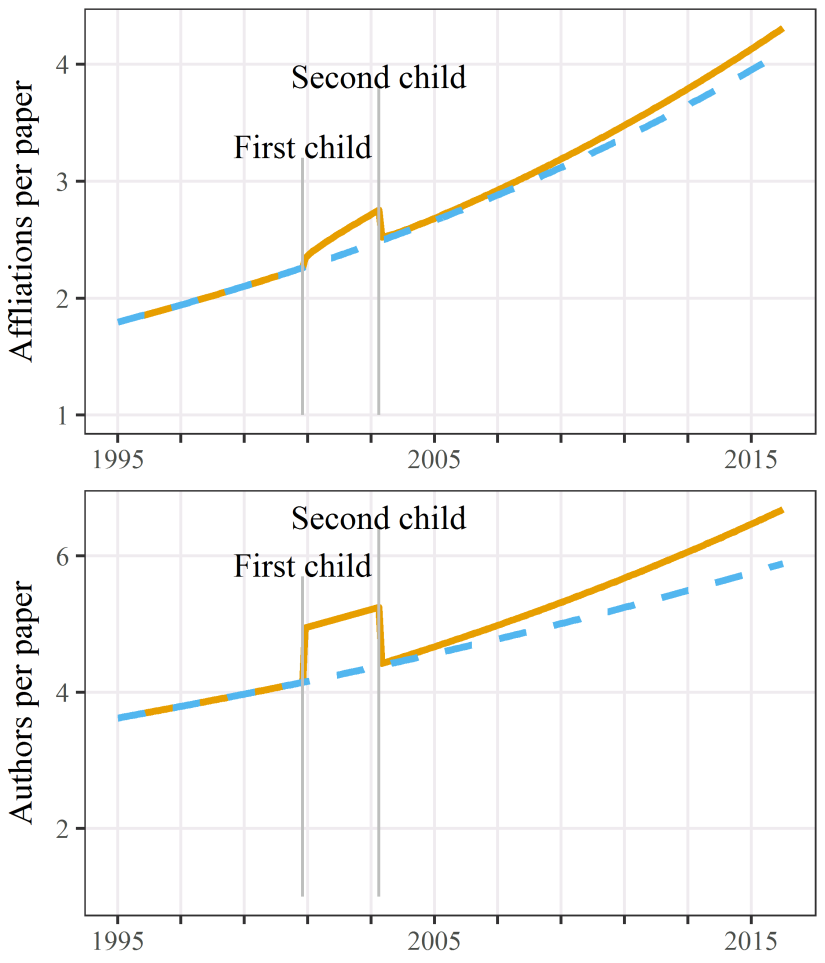


**Figure A10: Histograms of total number of citations and publications for all 160 sampled women.** The statistically influential participant 29 had the second highest number of total publications and citations.

**
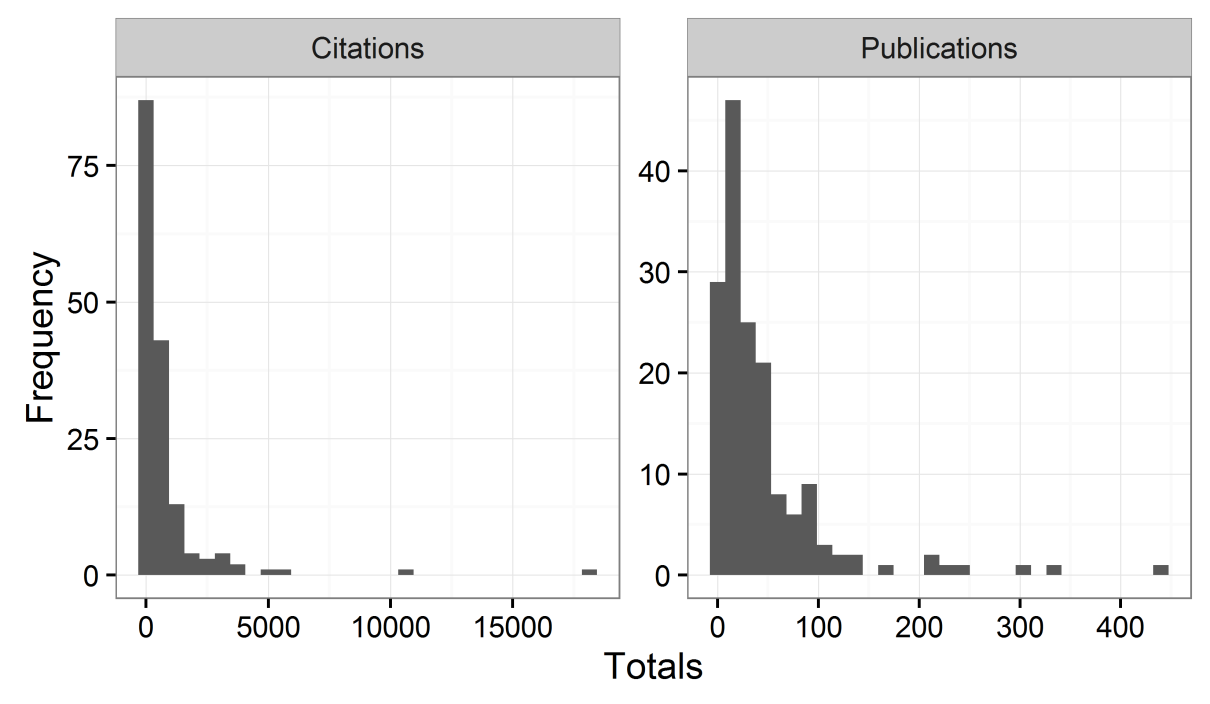
**

**Figure A11: Histograms of residuals for models of publication and citation numbers per year using two children.** The histograms are approximately unimodal with no large outliers.


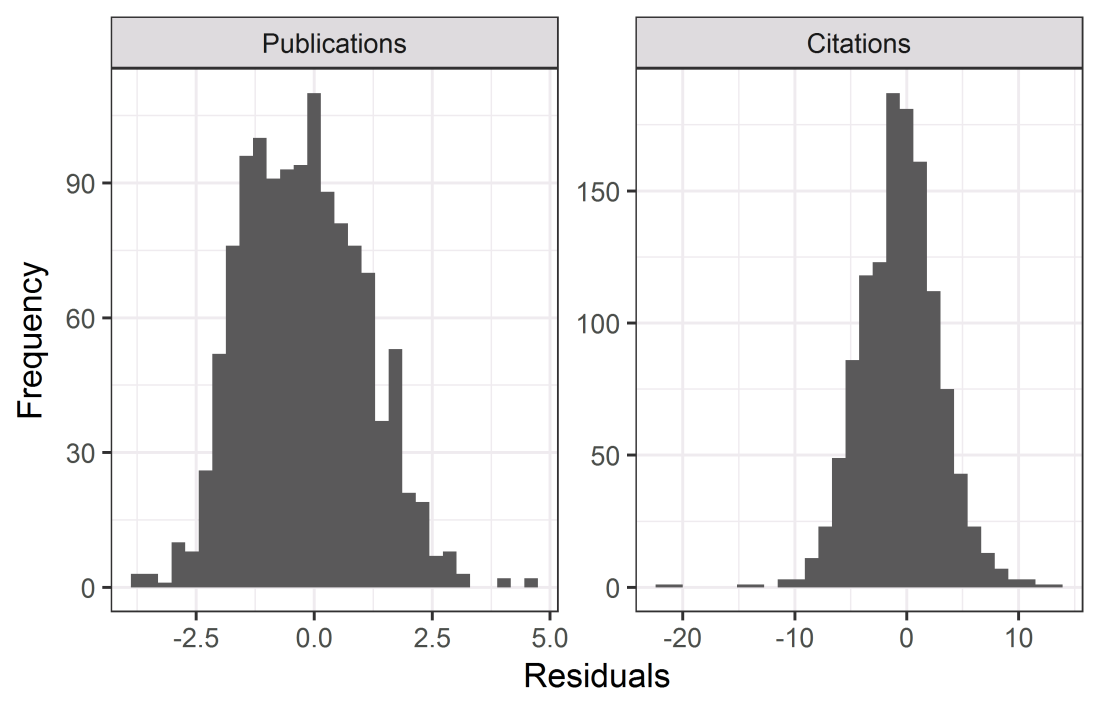


**Figure A12: Histograms of residuals for models of author, affiliation and country numbers per paper using two children.** The histograms are approximately unimodal with no large outliers.


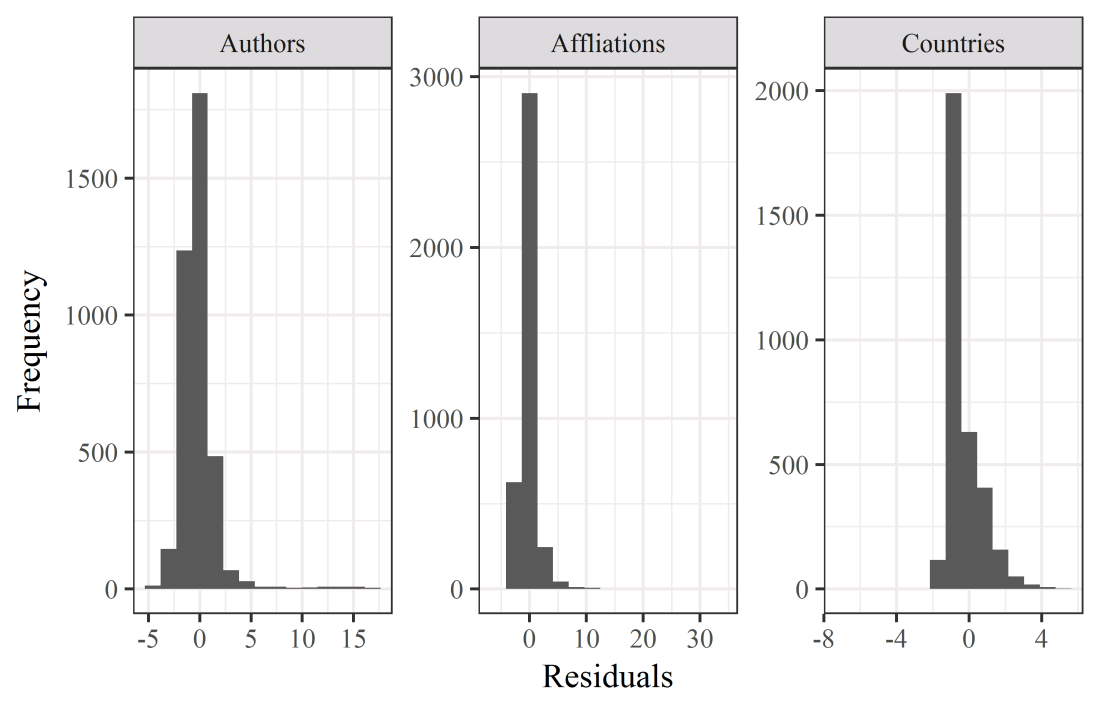


**Table A4: Akaike information criteria (AIC) for fractional polynomials for models with one or two children.** The model with a power of “None” has no time-dependent variables to model the impact of child care. The bolded AIC indicates the best combination of child numbers and fractional polynomial power. DNC = did not converge.

| Child numbers | Power | Citations | Publications | Author numbers | Affiliation numbers | Country not Australia | First author | Last author |
| --- | --- | --- | --- | --- | --- | --- | --- | --- |
| None | – | 22966.2 | 5277.7 | 27965.6 | 22573.9 | 5223.8 | 3993.9 | 3330.3 |
| One | –2.0 | DNC | DNC | DNC | DNC | DNC | DNC | DNC |
| One | –1.0 | DNC | DNC | DNC | DNC | DNC | DNC | DNC |
| One | –0.5 | DNC | DNC | DNC | DNC | DNC | 3995.7 | DNC |
| One | 0.0 | 22953.4 | 5280.4 | DNC | 22574.1 | DNC | DNC | DNC |
| One | 0.5 | 22941.9 | 5276.8 | 27958.1 | 22573.7 | **5221.4** | 3994.5 | 3332.6 |
| One | 1.0 | 22840.0 | 5277.3 | 27953.1 | 22573.7 | 5223.3 | 3995.0 | 3329.8 |
| One | 2.0 | 22569.0 | 5281.4 | 27956.4 | 22572.5 | 5224.7 | 3995.6 | 3325.9 |
| One | 3.0 | 22467.4 | 5281.5 | 27957.0 | 22565.8 | 5223.8 | 3995.6 | 3326.1 |
| One | None | 22957.6 | 5279.7 | 27956.5 | 22572.1 | 5222.8 | 3994.8 | 3331.3 |
| Two | –2.0 | DNC | DNC | DNC | DNC | DNC | DNC | DNC |
| Two | –1.0 | DNC | DNC | DNC | DNC | DNC | DNC | DNC |
| Two | –0.5 | DNC | DNC | DNC | DNC | DNC | DNC | DNC |
| Two | 0.0 | 22532.5 | DNC | DNC | DNC | DNC | DNC | DNC |
| Two | 0.5 | 22326.4 | 5256.7 | 27904.8 | **22553.1** | 5223.3 | DNC | DNC |
| Two | 1.0 | 22209.7 | DNC | **27901.7** | 22557.4 | DNC | 3991.9 | 3322.1 |
| Two | 2.0 | **22074.4** | 5226.4 | 27912.5 | 22563.0 | DNC | 3990.5 | **3317.7** |
| Two | 3.0 | 22082.3 | **5220.4** | DNC | 22557.0 | DNC | **3990.4** | DNC |
| Two | None | 22902.3 | 5275.6 | 27909.0 | 22572.1 | 5222.8 | 3994.8 | 3331.3 |

**Table A5: Estimated model parameters (log or logit scale) for women caring for two children after imputing non-responders.**

| **Outcome** | **Predictor** | **Mean** | **95% CI** |
| --- | --- | --- | --- |
| Authors | First child, time-fixed | 0.17 | –0.05 to 0.40 |
|  | Second child, time-fixed | –0.17 | –0.35 to 0.00 |
|  | First child, time-varying | 0.03 | –0.21 to 0.26 |
|  | Second child, time-varying | 0.08 | –0.11 to 0.27 |
| Affiliations | First child, time-fixed | 0.02 | –0.17 to 0.20 |
|  | Second child, time-fixed | –0.09 | –0.26 to 0.09 |
|  | First child, time-varying | 0.12 | –0.13 to 0.37 |
|  | Second child, time-varying | –0.06 | –0.27 to 0.15 |

**Output from generalised linear mixed models from R**

**Best model for publication counts**

Generalized linear mixed model fit by maximum likelihood (Laplace Approximation) ['glmerMod']

Family: poisson ( log )

Formula: papers ~ year.c + career.year.c + stan + baby.flag + stan2 + baby.flag2 + (1 | num)

Data: baby

AIC BIC logLik deviance df.resid

5220.4 5261.3 -2602.2 5204.4 1222

Scaled residuals:

Min 1Q Median 3Q Max

-2.6498 -0.9285 -0.2769 0.7392 10.7211

Random effects:

Groups Name Variance Std.Dev.

num (Intercept) 0.6751 0.8217

Number of obs: 1230, groups: num, 95

Fixed effects:

Estimate Std. Error z value Pr(>|z|)

(Intercept) 1.15645 0.11091 10.427 < 2e-16 ***

year.c 0.38714 0.09680 3.999 6.35e-05 ***

career.year.c 0.46491 0.09885 4.703 2.56e-06 ***

stan 0.53230 0.08003 6.651 2.91e-11 ***

baby.flag 0.11545 0.08418 1.372 0.1702

stan2 -0.53277 0.07214 -7.385 1.52e-13 ***

baby.flag2 -0.18024 0.08635 -2.087 0.0368 *

---

Signif. codes: 0 ‘***’ 0.001 ‘**’ 0.01 ‘*’ 0.05 ‘.’ 0.1 ‘ ’ 1

**Best model for citation counts**

Generalized linear mixed model fit by maximum likelihood (Laplace Approximation) ['glmerMod']

Family: poisson ( log )

Formula: citations ~ year.c + stan + Baby.flag + stan2 + Baby.flag2 + (1 | num)

Data: baby

AIC BIC logLik deviance df.resid

22074.4 22110.2 -11030.2 22060.4 1223

Scaled residuals:

Min 1Q Median 3Q Max

-20.1805 -2.3743 -0.5361 1.7525 15.2899

Random effects:

Groups Name Variance Std.Dev.

num (Intercept) 4.077 2.019

Number of obs: 1230, groups: num, 95

Fixed effects:

Estimate Std. Error z value Pr(>|z|)

(Intercept) 3.12808 0.20953 14.93 < 2e-16 ***

year.c 1.81374 0.01631 111.24 < 2e-16 ***

stan 0.39339 0.03951 9.96 < 2e-16 ***

Baby.flag -0.09525 0.02677 -3.56 0.000373 ***

stan2 -0.57602 0.03277 -17.58 < 2e-16 ***

Baby.flag2 -0.31237 0.02480 -12.60 < 2e-16 ***

---

Signif. codes: 0 ‘***’ 0.001 ‘**’ 0.01 ‘*’ 0.05 ‘.’ 0.1 ‘ ’ 1

**Best model for number of authors per paper**

Generalized linear mixed model fit by maximum likelihood (Laplace Approximation) ['glmerMod']

Family: poisson ( log )

Formula: n_auth ~ year.c + career.year.c + stan + Baby.flag + stan2 + Baby.flag2 + (1 | num)

Data: author.data

AIC BIC logLik deviance df.resid

27901.7 27951.7 -13942.8 27885.7 3837

Scaled residuals:

Min 1Q Median 3Q Max

-3.6052 -1.0098 -0.3971 0.3493 30.6256

Random effects:

Groups Name Variance Std.Dev.

num (Intercept) 0.1288 0.3589

Number of obs: 3845, groups: num, 95

Fixed effects:

Estimate Std. Error z value Pr(>|z|)

(Intercept) 1.71609 0.04864 35.28 < 2e-16 ***

year.c 0.22496 0.04390 5.12 2.99e-07 ***

career.year.c 0.03383 0.04710 0.72 0.473

stan 0.03066 0.05927 0.52 0.605

Baby.flag 0.27975 0.03916 7.14 9.04e-13 ***

stan2 0.06972 0.05005 1.39 0.164

Baby.flag2 -0.27538 0.03678 -7.49 6.99e-14 ***

---

Signif. codes: 0 ‘***’ 0.001 ‘**’ 0.01 ‘*’ 0.05 ‘.’ 0.1 ‘ ’ 1

**Best model for number of affiliations per paper**

Generalized linear mixed model fit by maximum likelihood (Laplace Approximation) ['glmerMod']

Family: poisson ( log )

Formula: n_affils ~ year.c + stan + Baby.flag + stan2 + Baby.flag2 + (1 | num)

Data: author.data

AIC BIC logLik deviance df.resid

22553.1 22596.9 -11269.6 22539.1 3838

Scaled residuals:

Min 1Q Median 3Q Max

-3.260 -0.964 -0.343 0.432 80.271

Random effects:

Groups Name Variance Std.Dev.

num (Intercept) 0.1746 0.4179

Number of obs: 3845, groups: num, 95

Fixed effects:

Estimate Std. Error z value Pr(>|z|)

(Intercept) 1.328187 0.065020 20.427 < 2e-16 ***

year.c 0.429159 0.027952 15.353 < 2e-16 ***

stan 0.289162 0.080533 3.591 0.00033 ***

Baby.flag 0.006558 0.067181 0.098 0.92223

stan2 -0.214818 0.069651 -3.084 0.00204 **

Baby.flag2 -0.117748 0.061816 -1.905 0.05681 .

---

Signif. codes: 0 ‘***’ 0.001 ‘**’ 0.01 ‘*’ 0.05 ‘.’ 0.1 ‘ ’ 1

**Best model for number of countries per paper**

Generalized linear mixed model fit by maximum likelihood (Laplace Approximation) ['glmerMod']

Family: poisson ( log )

Formula: n_not.australia ~ year.c + career.year.c + n_auth.c + baby.flag + stan + (1 | num)

Data: author.data

AIC BIC logLik deviance df.resid

5221.4 5264.3 -2603.7 5207.4 3377

Scaled residuals:

Min 1Q Median 3Q Max

-4.7727 -0.5713 -0.3784 -0.2189 10.5882

Random effects:

Groups Name Variance Std.Dev.

num (Intercept) 0.8926 0.9448

Number of obs: 3384, groups: num, 94

Fixed effects:

Estimate Std. Error z value Pr(>|z|)

(Intercept) -1.52976 0.17422 -8.78 <2e-16 ***

year.c 0.20863 0.14062 1.48 0.1379

career.year.c 0.32570 0.15135 2.15 0.0314 *

n_auth.c 0.23770 0.00644 36.91 <2e-16 ***

baby.flag -0.04979 0.16401 -0.30 0.7615

stan -0.25913 0.14145 -1.83 0.0670 .

---

Signif. codes: 0 ‘***’ 0.001 ‘**’ 0.01 ‘*’ 0.05 ‘.’ 0.1 ‘ ’ 1

**Best model for first author**

Generalized linear mixed model fit by maximum likelihood (Laplace Approximation) ['glmerMod']

Family: binomial ( logit )

Formula: first.author ~ year.c + career.year.c + n_auth.c + Baby.flag +

stan + Baby.flag2 + stan2 + (1 | num)

Data: order

AIC BIC logLik deviance df.resid

3990.4 4046.7 -1986.2 3972.4 3828

Scaled residuals:

Min 1Q Median 3Q Max

-2.301 -0.605 -0.374 0.732 51.413

Random effects:

Groups Name Variance Std.Dev.

num (Intercept) 0.5333 0.7302

Number of obs: 3837, groups: num, 95

Fixed effects:

Estimate Std. Error z value Pr(>|z|)

(Intercept) -0.89133 0.14217 -6.269 3.62e-10 ***

year.c -0.03052 0.11087 -0.275 0.7831

career.year.c -0.92286 0.11855 -7.784 7.01e-15 ***

n_auth.c -0.38435 0.06874 -5.591 2.26e-08 ***

Baby.flag 0.29345 0.18010 1.629 0.1032

stan 0.28590 0.18903 1.512 0.1304

Baby.flag2 -0.27304 0.18172 -1.503 0.1330

stan2 -0.42030 0.18383 -2.286 0.0222 *

---

Signif. codes: 0 ‘***’ 0.001 ‘**’ 0.01 ‘*’ 0.05 ‘.’ 0.1 ‘ ’ 1

**Best model for last author**

Generalized linear mixed model fit by maximum likelihood (Laplace Approximation) ['glmerMod']

Family: binomial ( logit )

Formula: last.author ~ year.c + career.year.c + n_auth.c + baby.flag +

stan + baby.flag2 + stan2 + (1 | num)

Data: order

AIC BIC logLik deviance df.resid

3317.7 3374.0 -1649.9 3299.7 3828

Scaled residuals:

Min 1Q Median 3Q Max

-2.035 -0.505 -0.277 -0.003 188.649

Random effects:

Groups Name Variance Std.Dev.

num (Intercept) 0.3525 0.5938

Number of obs: 3837, groups: num, 95

Fixed effects:

Estimate Std. Error z value Pr(>|z|)

(Intercept) -1.92265 0.14866 -12.933 < 2e-16 ***

year.c -0.32418 0.10975 -2.954 0.00314 **

career.year.c 0.93754 0.11841 7.917 2.42e-15 ***

n_auth.c -2.14888 0.12894 -16.666 < 2e-16 ***

baby.flag -0.09918 0.22465 -0.441 0.65887

stan -0.57861 0.20601 -2.809 0.00498 **

baby.flag2 0.44025 0.21533 2.045 0.04090 *

stan2 0.36514 0.18940 1.928 0.05387 .

---

Signif. codes: 0 ‘***’ 0.001 ‘**’ 0.01 ‘*’ 0.05 ‘.’ 0.1 ‘ ’ 1
