# Supplementary material for: The impact of caring for children on women’s research output: A retrospective cohort study
Source: PLoS One. 2019 Mar 21;14(3):e0214047. doi: 10.1371/journal.pone.0214047 (PMC6428253; doi:10.1371/journal.pone.0214047)
Supplement: S3 Appendix — (DOCX) [file pone.0214047.s004.docx]

**Appendix S3 – Participants’ comments**

1. Hi there, I am no longer research active but as I have not had children to care for, this has had no impact on my research productivity. [Participant 67]
2. I think I made much better life choices because I was caring for children. My hours at work were so few, and the opportunity cost of being away from my kids so high, that I had to treat every working hour as something precious and scarce. As a result, I became much better at doing only work that really mattered to me and had a real potential to make a difference. [Participant 11]
3. Working in the non-for-profit sector means that publishing work comes second to other organization priorities. Publishing happens in researchers down time or own time. Having children at home and/or working part time makes this very difficult. Good luck with the research. [Participant 65]
4. I did not work in academia or as a researcher when my children were very young. While I think that the focus on caring is very important other factors are also important, for example, career changes, the slowing of research productivity while undertaking a PhD, being employed in a teaching focused position etc. I think you would need to gather much more information from the individual themselves in order to ascertain how caring for children impacts on research productivity. [Participant 93]
5. I wasn't sure how to answer the final column. The question seems to assume that you take time of the workforce to have a baby? i moved into the workforce while caring. Also, no capacity to provide further context: i was a single mother and one of the children had a disability - requires ongoing care. [Participant 91]
6. The answer options to the question about time to return to work do not offer the opportunity to give accurate information. I tried to put 62 months in because i took 5 years away from work and this was rejected. [Participant 63]
7. In my experience, it is hard to maintain research outputs while caring for children-mainly because if you are not present in the workplace your contribution to the intellectual side of papers and grants gets forgotten, and you are not asked sometime also-and papers and grants get submitted without you being included. Also, maintaining employment and entitlements while only working casual or part time is very difficult-in a very insecure employment environment anyway. Often if feels like people think when you have a child you lose some of your brain-and can no longer perform at a high level-so you are overlooked for senior positions. Both men and women can also be very uncompromising and judgemental in the research environment about juggling caring and work requirements. And often women have to over-compensate by working long hours late at night to be seen to be contributing sufficiently to warrant still being seen as a committed and active researcher. [Participant 26]
8. Whilst I have not yet cared for children, I do anticipate doing so in the future. As an early-career research I am concerned about the potential impact this may have on future research productivity. Best wishes with your research. [Participant 9]
9. I do not have any carers responsibilities to date. However, planning for children weighs heavily on the mind, particularly under the pressure of writing and submitting grant applications where you propose you will conduct independent research over a given year or of only being employed on short-term research contracts. [Participant 36]
10. It is a matter of balancing work and home expectations and getting assistance in caring for children and the home. It is never going to be easy. [Participant 39]
11. It is curious that only one question is asked to quantify the impact of caring for children on womens research productivity, which comes down to so much more than a relatively brief period of maternity leave..... will be interesting to see the outcome of the study. [Participant 4]
12. Parenting definitely impacts on research productivity- great that you are trying to quantify this. [Participant 88]
13. I think it is worth mentioning I am in a same sex relationship, and was able to return to full time work within 3 months of the arrival of my daughter as my partner (also female) took 12 months maternity leave from her research role; she returned to work 3 days a week after 12 months and 4 days per week after 24 months. [Participant 24]
14. Wonder why you are not taking age and caring for parents or other caring into account. [Participant 6]
15. I appreciate that your study is focusing on caring for children, however, have you considered that women are also often the primary carers for others eg parents etc.? Would it be worth measuring that aspect as well as it has potential to confound your results? [Participant 8]
16. From my experience, the impact on productivity of caring for children is complex. Quantifying to research production to papers published is one valuable metric, but pathways and opportunities to publish is also affected by caring for children. All this is impacted by the choices women make about when they return to work and in what capacity. Thanks. [Participant 86]
17. Slowed my progress and career development though don't think it has affected my competitiveness overall. [Participant 22]
18. Difficult :) [Participant 72]
19. I was studying before I went back to work hence the long break of 24 months. [Participant 44]
20. I don't know how anyone could manage to care for children while working full time without a lot of help. I have never done it but I can imagine it would be next to impossible. [Participant 14]
21. I was heavily reliant on good and accessible child care and needed well paid work to be able to afford it. I still feel guilty about working when my kids were so young. [Participant 48]
22. Biggest issue is balancing parental responsibilities with professional responsibilities. Net effect is attempting to do everything but feeling like you never do anything properly. [Participant 92]
23. I think it is important to keep in mind even though children are in childcare or school, there is still a large impact on the productivity. [Participant 27]
24. I thought you were also interested in women who have not cared for children? [Participant 61]
25. I returned to work for only 8 hrs/week with my second child and had worked part time (0.5 eft) with my first child. [Participant 21]
26. simply knowing how long it took between commencing care and returning to work is of limited usefulness. there are so many factors that influence work patterns when care for chldren is also occurring. without collecting data on other factors that may contribute how will this study really contribute anything meaningful? [Participant 29]
27. My first daughter was born in the US and I was in the middle of doing my PhD. In the US they return to work much sooner than Aus - and as was doing my research I felt had to get back into it very quickly. The next two were born in Australia and I took longer - but now people take much longer still. I don't know if I'd do it differently if I had my time over. Putting a career on hold can be very career-limiting, but being distracted by work when they are babies is not good either. Finding the right child care is only part of the battle - finding peace with juggling things or choosing one over the other is also a big part of it. I have had great opportunities in my career in part because of the PhD I did finish despite a newborn at the time. I think my 3 daughters have taken it all in (the fact that I am their mum and I have a career as well - all those pluses and minuses are part of their childhood experience). I also believe that children who are in at least part-time childcare from an early age, seem to have more resilience later in life. Learning to solve one's own problems may be something that comes with not having a mum on hand every minute. [Participant 34]
28. Extremely difficult to juggle competing demands. While maternity leave is recognised & accepted in assessment of research outputs, ongoing Childcare responsibilities are not. [Participant 30]
29. A great research topic. It is definitely a challenge caring for children, working and publishing papers. [Participant 58]
30. It is certainly stressful both mentally and physically. Even though the kids went to day care or school during the time I worked, it was like starting a second shift at the end day when I picked up them from afterschool care and daycare and brought them home. You were exhausted at the end of the day but you had to bite the "bullet" of this second shift: getting a healthy dinner ready for the kids, washing up in the kitchen, getting them ready for bed and getting things ready for the next day as you had to rise early everyday. Having good health and partners' support and help enable you to cope reasonably well. Being able to work flexible times was a great help. Caring child reduced one's opportunity to participate fully in academic life and miss things like seminars taking place after 5pm and you could just go to as many conferences as you wished to attend. It might have limited my opportunities for involving in research grant applications. [Participant 53]
31. The questions/answers in this short survey are not relevant to my time as a researcher. I was working in the clinical field whilst my children were living at home and started research many years later. I have decided to submit the survey but really do not believe this response will add validity to your data analysis. [Participant 90]
32. Child comes first always; this means missed opportunities for travel to research conferences as I needed to stay home. [Participant 41]
33. caring for elderly parents is a consideration also. [Participant 55]
34. I was working in a non-research role in Government between 2011-2013 which meant research publications were not a priority at that time. I think the survey needs to account for the job/role a person has during the time you are assessing their publications. I have just returned to work from 6 months maternity leave this week - hence my late reply to this survey- hope it is still helpful though! [Participant 35]
